# Gut-innervating TRPV1+ Neurons Drive Chronic Visceral Pain via Microglial P2Y12 Receptor

**DOI:** 10.1016/j.jcmgh.2021.12.012

**Published:** 2021-12-24

**Authors:** Manon Defaye, Nasser S. Abdullah, Mircea Iftinca, Ahmed Hassan, Francina Agosti, Zizhen Zhang, Melissa Cumenal, Gerald W. Zamponi, Christophe Altier

**Affiliations:** 1 Department of Physiology and Pharmacology, Calgary, Alberta, Canada; 2 Inflammation Research Network-Snyder Institute for Chronic Diseases, Calgary, Alberta, Canada; 3 Alberta Children's Hospital Research Institute, Cumming School of Medicine, Calgary, Alberta, Canada; 4 Hotchkiss Brain Institute, Cumming School of Medicine, University of Calgary, Calgary, Alberta, Canada

**Keywords:** Microglia, P2RY12, TRPV1 Neurons, Visceral Pain, AP, action potential, ATP, adenosine triphosphate, BSA, bovine serum albumin, CGRP, calcitonin gene-related peptide, CNO, Clozapine-N-oxide, CNS, central nervous system, CRD, colorectal distension, DREADD, designer receptors exclusively activated by designer drugs, DRG, dorsal root ganglia, DSS, dextran sodium sulfate, ENS, enteric nervous system, GFRα2, Glial cell-derived neurotrophic factor family receptor alpha 2, IBD, inflammatory bowel disease, IBS, irritable bowel syndrome, IFN-γ, interferon-γ, IL, interleukin, i.p., intraperitoneally, OCT, optimal cutting temperature, PBS, phosphate buffered saline, PFA, paraformaldehyde, RT, room temperature, TNF-α, tumor necrosis factor-α, TRPV1, transient receptor potential vanilloid member 1, VHS, visceral hypersensitivity, VMR, visceromotor response

## Abstract

**Background & Aims:**

Chronic abdominal pain is a common symptom of inflammatory bowel diseases (IBDs). Peripheral and central mechanisms contribute to the transition from acute to chronic pain during active disease and clinical remission. Lower mechanical threshold and hyperexcitability of visceral afferents induce gliosis in central pain circuits, leading to persistent visceral hypersensitivity (VHS). In the spinal cord, microglia, the immune sentinels of the central nervous system, undergo activation in multiple models of VHS. Here, we investigated the mechanisms of microglia activation to identify centrally acting analgesics for chronic IBD pain.

**Methods:**

Using Designer Receptors Exclusively Activated by Designer Drugs (DREADD) expressed in transient receptor potential vanilloid member 1-expressing visceral neurons that sense colonic inflammation, we tested whether neuronal activity was indispensable to control microglia activation and VHS. We then investigated the neuron-microglia signaling system involved in visceral pain chronification.

**Results:**

We found that chemogenetic inhibition of transient receptor potential vanilloid member 1^+^ visceral afferents prevents microglial activation in the spinal cord and subsequent VHS in colitis mice. In contrast, chemogenetic activation, in the absence of colitis, enhanced microglial activation associated with VHS. We identified a purinergic signaling mechanism mediated by neuronal adenosine triphosphate (ATP) and microglial P2Y12 receptor, triggering VHS in colitis. Inhibition of P2RY12 prevented microglial reactivity and chronic VHS post-colitis.

**Conclusions:**

Overall, these data provide novel insights into the central mechanisms of chronic visceral pain and suggest that targeting microglial P2RY12 signaling could be harnessed to relieve pain in patients with IBD who are in remission.

## Graphical abstract

**Figure:**
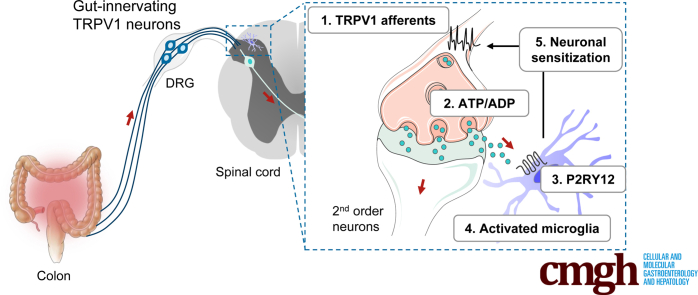

**Summary**

Chronic abdominal pain is one of the most debilitating symptoms of inflammatory bowel disease. Microgliosis in central pain circuits drives persistent visceral hypersensitivity, but how spinal microglia are activated remains unclear. Adenosine triphosphate released from transient receptor potential vanilloid member 1^+^ visceral afferents drives microglial reactivity via P2RY12 in colitis-induced visceral pain.

Inflammatory bowel diseases (IBDs), including Crohn’s disease and ulcerative colitis, are chronic relapsing and remitting inflammatory diseases associated with abdominal pain.^1^ Although up to 90% of patients with active IBD have abdominal pain,^2^^,^^3^ a subset of patients (20%–60%) continue to experience episodic or persistent intractable pain, including visceral hypersensitivity (VHS), despite being in endoscopic remission,^2^^,^^4^ suggesting a high level of plasticity in the afferent pain pathway during inflammatory phases of IBD.

Although molecular and functional changes in gut nociceptors enhance nociceptive input at the periphery,^5^ the central mechanisms of sensitization along the entire neural axis remain largely unknown. Eight major cell types have been identified in the mouse spinal cord.^6^ Among them, microglia are activated in the spinal dorsal horn following nerve injury or inflammation, highlighting the importance of dorsal root ganglia (DRG) neuron-microglia interactions in pathological pain. Enhanced microglia reactivity contributes to persistent pain in several preclinical pain models, including peripheral and spinal injury, peripheral inflammation, bone cancer pain, and colitis.^7^, ^8^, ^9^, ^10^, ^11^ During active colitis, activated microglia produce a large panel of proinflammatory mediators including interleukin-6 (IL-6), IL-1β, tumor necrosis factor-α (TNF-α), and brain-derived neurotrophic factor (BDNF), which contribute to neuronal sensitization.^11^, ^12^, ^13^ However, microglial activation persists during the recovery phase of colitis in which animals experience VHS.^14^ Accordingly, we reported that VHS is mediated by microglial activation in dextran sodium sulfate (DSS)-induced colitis. We identified granulocyte-colony stimulating factor as a central factor sensitizing visceral afferents that express the transient receptor potential vanilloid member 1 (TRPV1), which ultimately induce visceral sensitization.^15^

TRPV1-expressing neurons, prominently in peptidergic classes, make up 65% to 95% of gut innervating afferents depending on the DRG level.^16^ TRPV1 is a nonselective cation channel responding to a variety of noxious stimuli including inflammatory mediators produced by innate-immune cells, and damage-associated molecular patterns released in the inflamed tissue.^17^ Furthermore, growing evidence suggests that sensitization of TRPV1 mediates VHS during resolution of colitis,^18^ a well-defined process that likely contributes to VHS in preclinical models of colitis and patients with irritable bowel syndrome (IBS) or quiescent IBD.^19^, ^20^, ^21^

Here we investigated whether VHS results from the direct communication between sensitized TRPV1^+^ visceral nociceptors and spinal microglia. We found that chemogenetic inhibition of TRPV1^+^ neurons reduced colitis-induced microglia activation and VHS, whereas chemogenetic activation without colitis, was sufficient to enhance microglial reactivity leading to VHS. We next determined the mechanism by which TRPV1^+^ neurons promote microglia activation in response to neuronal activity or experimental DSS colitis.

## Results

### Generation of TRPV1-DREADD Mice

**Figure 1:**
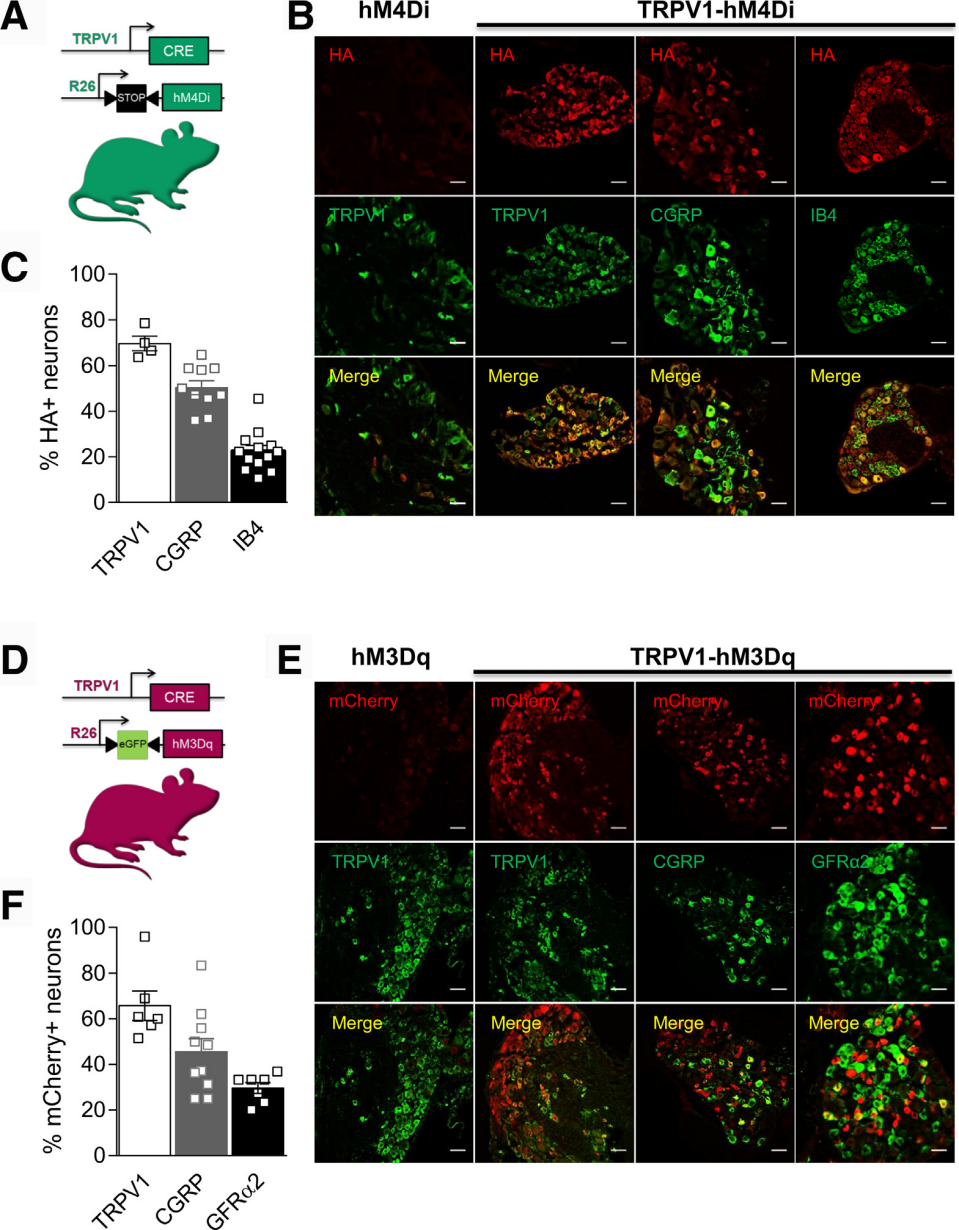
**hM4Di and hM3Dq expression in TRPV1**^**+**^**DRG neurons.***A*, Schematic representation of the transgenic TRPV1-hM4Di mouse design. *B*, HA-tagged hM4Di expression was confirmed by HA immunostaining in different subsets of neurons from T10-L1 and L6-S1 levels. Scale bars equal 50 μm. *C*, Dot plot showing the percentage of peptidergic (TRPV1 and CGRP) and non-peptidergic (IB4) neurons that co-express HA-tagged hM4Di in DRG from TRPV1-hM4Di mice (n = 3). *D*, Schematic representation of the transgenic TRPV1-hM3Dq mouse design. *E*, mCherry-tagged hM3Dq visualization in different subsets of neurons from T10-L1 and L6-S1 levels. Scale bars equal 50 μm. *F*, Dot plot showing the percentage of peptidergic (TRPV1 and CGRP) and non-peptidergic (GFRα2) neurons co-expressing hM3Dq-mCherry in DRG from TRPV1-hM3Dq mice (n = 3).

To assess the contribution of TRPV1^+^ visceral afferents in microglial activation, we generated Designer Receptors Exclusively Activated by Designer Drugs (DREADD) mice in which TRPV1^+^ nociceptors are chemically inhibited (TRPV1-hM4Di) or activated (TRPV1-hM3Dq) by the inert ligand clozapine-N-oxide (CNO).^22^ As reported by Saloman et al,^23^ the TRPV1-hM4Di mice were produced by breeding mice containing an hM4Di-HA-mCitrine sequence in the ROSA26 locus^24^ and mice expressing Cre recombinase downstream of the TRPV1 promoter^25^ (Figure 1*A*). TRPV1-hM3Dq mice were produced by crossing the TRPV1-Cre mice with the floxed hM3Dq mice that contain an inverted hM3Dq-mCherry sequence in the ROSA26 locus downstream of a FLEx switch containing an eGFP cassette^26^ (Figure 1*D*). When assessing the efficiency of Cre recombinase and the expression of hM4Di and hM3Dq in TRPV1^+^ neurons (HA vs mCherry) (Figure 1*B*, 1*E*), we found that 75% of TRPV1^+^ neurons and 50% of the calcitonin gene-related peptide (CGRP)-labeled neurons expressed HA or mCherry in TRPV1-hM4Di and TRPV1-hM3Dq mice, respectively (Figure 1*C*, 1*F*). However, as reported in previous studies,^25^ a small proportion of nonpeptidergic neurons immunopositive for the isolectin B4 or the glial cell-derived neurotrophic factor family receptor alpha 2 (GFRα2) expressed TRPV1 and thus the DREADD receptors (Figure 1*C*, 1*F*). Together, our data show that TRPV1-DREADD mice express the receptor efficiently in a large proportion of small peptidergic TRPV1^+^ neurons.

**Figure 2:**
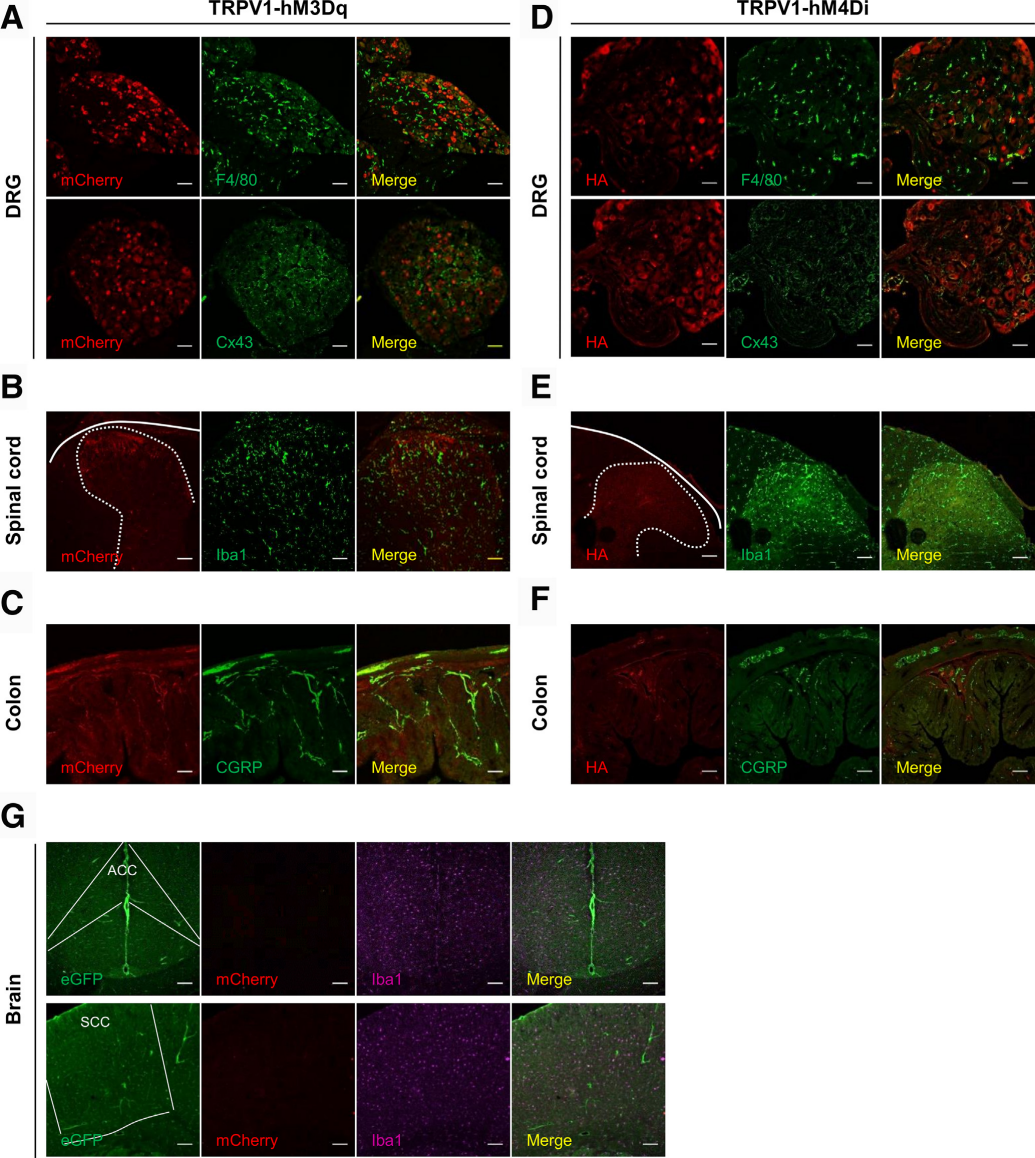
**hM3Dq-mCherry is not expressed in non-neuronal cells and in the ENS.** The absence of hM3Dq and hM4Di expression in non-neuronal cells and ENS was confirmed by staining with an anti F4/80 (macrophages) and Cx43 (satellite glial cells) antibodies in DRG (*A* and *D*, respectively); Iba1 antibody (microglia) in spinal cord (*B* and *E*, respectively), and CGRP antibody in the colon (*C* and *F*, respectively). *G*, hM3Dq expression was not detected in the anterior cingulate cortex and the somatosensory cortex in the brain. Scale bars 50 μm.

Previous studies have suggested the expression of TRPV1 in non-neuronal cells and in the enteric nervous system (ENS).^27^, ^28^, ^29^, ^30^ We thus assessed DREADD expression in satellite glial cells, microglia, and macrophages centrally and at the periphery. Expression of hM3Dq and hM4Di was not found in macrophages (F4/80) and satellite glial cells (Connexin 3, Cx43) of the DRGs (Figure 2*A*, 2*D*). Iba-1 immunostaining for microglia showed the absence of hM3Dq expression in non-neuronal cells of the spinal cord (Figure 2*B*, 2*E*). Moreover, colocalization of both the mCherry-tagged hM3Dq, and CGRP expressing fibers in the colonic mucosa, confirmed the absence of hM3Dq expression in non-neuronal cells and in the ENS of the colonic wall (Figure 2*C*, 2*F*). Although we could image a weak HA signal in the sensory fibers projecting to the spinal cord or the gut, the hM4Di receptor was not found in the soma of non-neuronal cells of the CNS (Figure 2*E*) and in the colon (Figure 2*F*). Finally, we did not observe DREADD expression in the anterior cingulate cortex and the somatosensory cortex, which are key brain areas involved in the perception of pain^31^ (Figure 2*G*).

**Figure 3:**
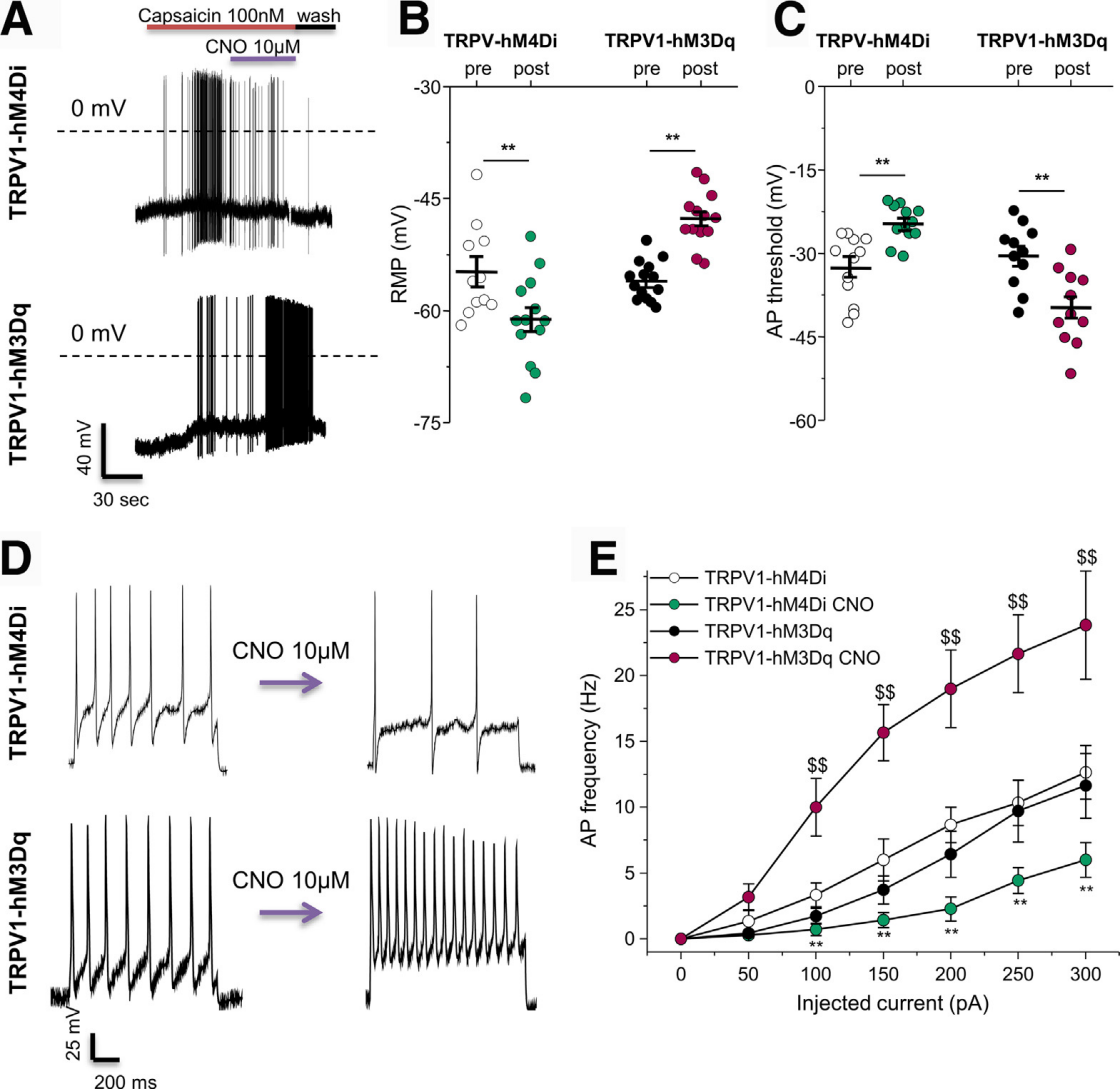
**Effect of CNO on TRPV1**^**+**^**DRG neuron excitability.***A*, Representative traces showing the effect of CNO (10 μM) on capsaicin-induced (100 nM) depolarization and AP firing in a DRG neurons from TRPV1-hM4Di or TRPV1-hM3Dq mice. Note the hyperpolarization and inhibition of AP firing in TRPV1-hM4Di, but the depolarization and increased AP firing in TRPV1-hM3Dq neurons. *B*, CNO exposure hyperpolarizes or depolarizes the resting membrane potential (RMP) of TRPV1-hM4Di and TRPV1-hM3Dq neurons, respectively (TRPV1-hM4Di: −56.6 ± 2.47 mV vs −64.5 ± 2.28 mV, n = 9; TRPV1-hM3Dq: −56.01 ± 0.7 mV vs −47.7 ± 0.99 mV, n = 14). Statistical analysis was performed using the paired *t* test (∗∗*P* < .01). *C*, CNO decreases or increases the action potential threshold of capsaicin-responsive TRPV1-hM4Di and TRPV1-hM3Dq neurons, respectively (TRPV1-hM4Di: −32.6 ± 1.7 mV vs −24.7 ± 0.95 mV, n = 12; TRPV1-hM3Dq: −30.4 ± 1.7 mV vs −39.7 ± 2.0 mV, n = 11). Statistical analysis was performed using the paired *t* test (∗∗*P* < .01). *D*, Representative traces of AP firing evoked by 200 pA of injected current (1 second pulse) in the absence and presence of CNO (10 μM) in TRPV1-hM4Di (*top*) and TRPV1-hM3Dq (*bottom*) neurons. *E*, The AP frequency evoked by increasing the amount of injected current was greater in TRPV1-hM3Dq but lower in TRPV1-hM4Di neurons (TRPV1-hM4Di: 12.7 ± 2.04 Hz vs 6.0 ± 1.32 Hz, n = 6; TRPV1-hM3Dq: 11.6 ± 2.5 Hz vs 23.8 ± 4.1 Hz, n = 7, elicited by 300 pA of current). Statistical analysis was performed using 2-way analysis of variance followed by the Bonferroni post hoc test (∗∗*P* < .05 vs TRPV1-hM4Di; ^$$^*P* < .01 vs TRPV1-hM3Dq). Three independent experiments were performed.

To validate the functional expression of the DREADDs in TRPV1^+^ neurons, we tested the effect of CNO on neuronal excitability. Current-clamp experiments were performed on dissociated DRG neurons from TRPV1-hM4Di and TRPV1-hM3Dq mice. CNO exposure (10 μM) resulted in a decrease in action potential (AP) firing in TRPV1^+^ neurons expressing hM4Di following capsaicin-induced depolarization (Figure 3*A*, *top*). In contrast, CNO perfusion increased the AP firing of TRPV1-hM3Dq neurons (Figure 3*A*, *bottom*). Furthermore, in capsaicin-responsive neurons, CNO induced a hyperpolarization of the membrane potential (Figure 3*B*) and a greater AP threshold (Figure 3*C*) in TRPV1-hM4Di neurons, whereas TRPV1-hM3Dq neurons displayed a more depolarized resting membrane potential (Figure 3*B*) and a lower AP threshold (Figure 3*C*). Finally, the AP frequency evoked by an ascending ramp of injected current was lower in TRPV1-hM4Di and greater in TRPV1-hM3Dq neurons after CNO perfusion (Figure 3*D*, 3*E*). These data confirm the functional expression of both hM4Di and hM3Dq in TRPV1^+^ neurons.

### Chemogenetic Inhibition of TRPV1^+^ Neurons Prevents Microglial Activation in DSS-induced Colitis

**Figure 4:**
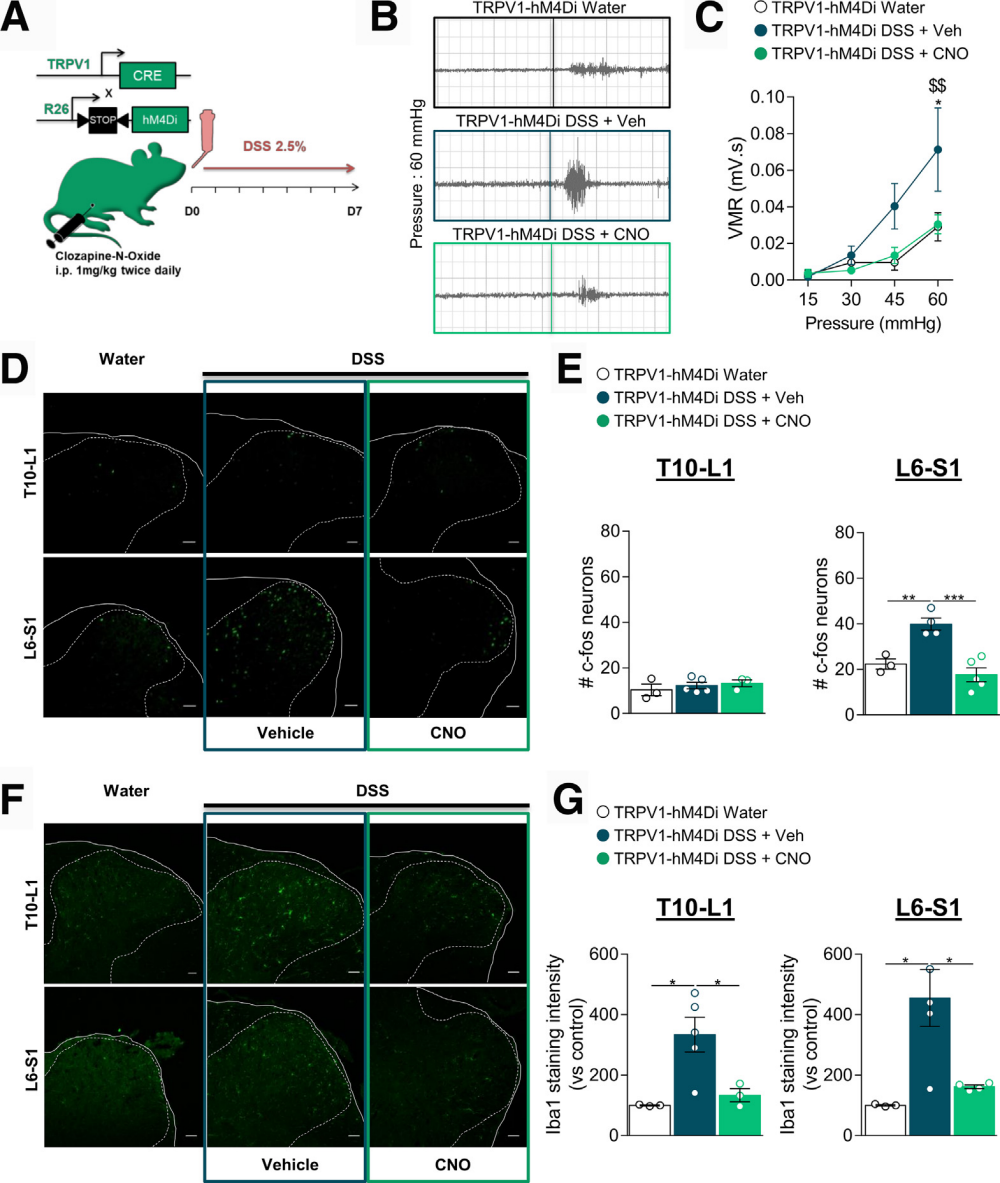
**Inhibition of TRPV1**^**+**^**neurons prevents VHS, neuronal and microglial activation in the spinal cord of colitis mice.***A*, Schematic representation of the experimental design. Colitis was induced by adding 2.5% DSS in the drinking water (water as control) of TRPV1-hM4Di mice while receiving an i.p. injection of CNO twice daily (1 mg/kg, DSS + CNO) or vehicle (DSS + Veh) for 7 days. *B*, Representative traces of CRD test showing the effect of chronic CNO on DSS-induced VHS in TRPV1-hM4Di mice. *C*, VMR to colorectal distension in DSS colitis mice after treatment with CNO or Veh (Water ( n = 4), DSS + Veh ( n = 7), or DSS + CNO ( n = 6)). Statistical analysis was performed using 2-way analysis of variance followed by Tukey post hoc test (∗*P* < .05 vs water; ^$$^*P* < .01 vs DSS + CNO). *D*, Representative confocal images of c-Fos positive neurons immunostained in the thoracolumbar (T10–L1) and lumbosacral (L6–S1) spinal cord sections. Scale bar = 50 μm. *E*, Dot plot showing the average number of c-Fos positive neurons in the spinal dorsal horn of water ( n = 3), DSS + Veh ( n = 4–5), and DSS + CNO ( n = 3–5) treated mice. Statistical analysis was performed using 1-way analysis of variance followed by the Tukey post hoc test (∗∗*P* < .01; ∗∗∗*P* < .001). *F*, Representative confocal images of microglia immunoreactive to Iba-1 quantified in the T10–L1 and L6–S1 spinal cord sections. Scale bar = 50 μm. *G*, Dot plot showing the intensity of Iba-1 immunostaining in the spinal dorsal horn of water ( n = 3), DSS + Veh ( n = 5), and DSS + CNO ( n = 3–4) treated mice. Statistical analysis was performed using 1-way analysis of variance followed by the Tukey post hoc test (∗*P* < .05). Three independent experiments were performed.

**Figure 5:**
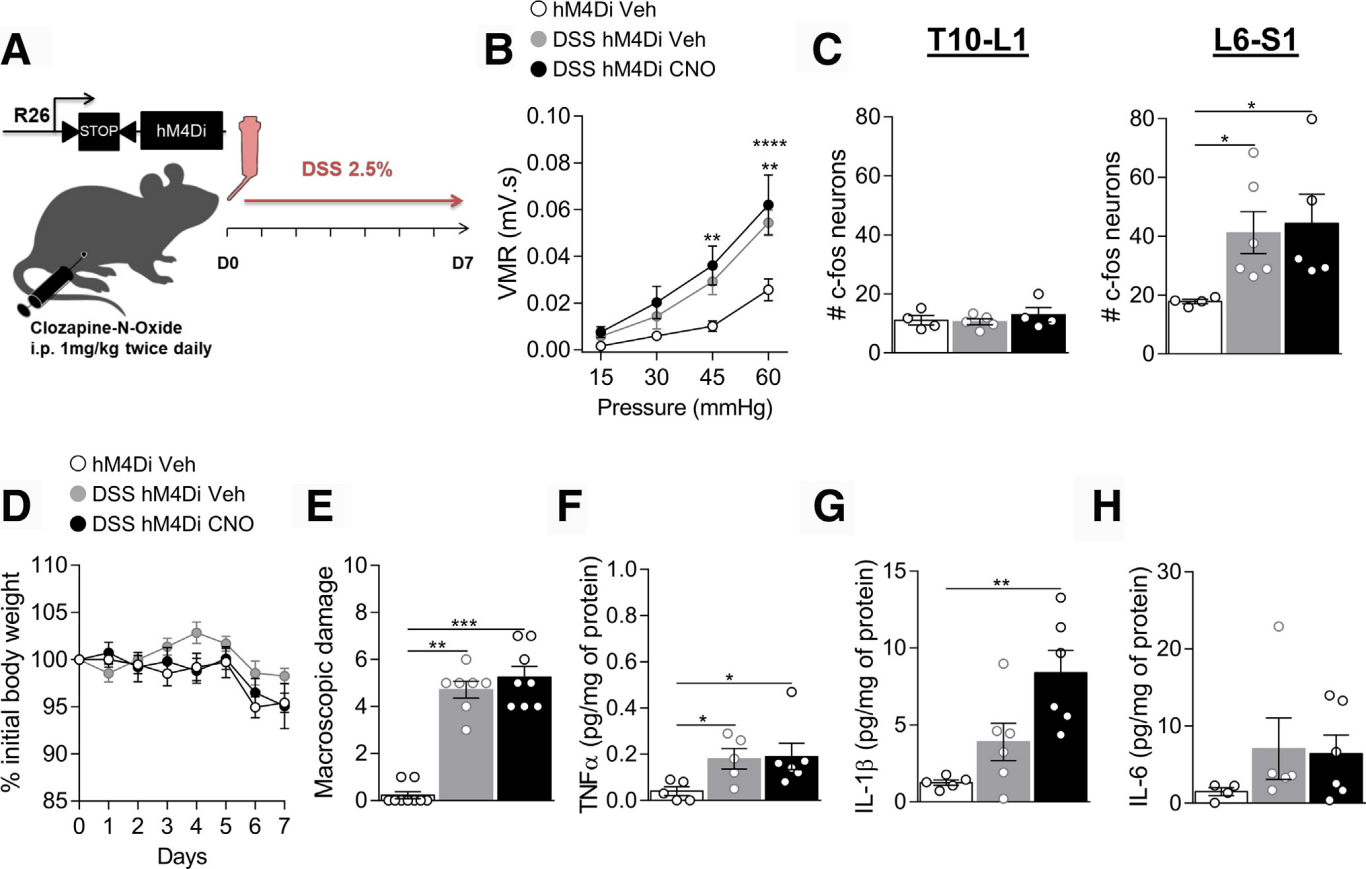
**CNO has no effect on hM4Di littermate controls during colitis.***A*, Schematic representation of the experimental design. Colitis was induced by adding 2.5% DSS in the drinking water (water as control) of the hM4Di mice while receiving twice daily an i.p. injection of vehicle (DSS + Veh) as control or CNO 1 mg/kg (DSS + CNO). *B*, VMR to colorectal distension after treatment with water ( n = 9), DSS + Veh ( n = 7), or DSS + CNO ( n = 8). Statistical analysis was performed using 2-way analysis of variance followed by Tukey post hoc test (∗∗*P* < .01; ∗∗∗∗*P* < .0001 vs water). *C*, Dot plot showing the average number of c-Fos positive neurons in the spinal dorsal horn of water ( n = 4), DSS + Veh ( n = 5–6), or DSS + CNO ( n = 4–5) treated mice. Statistical analysis was performed using Kruskal-Wallis followed by the Dunn post hoc test (∗*P* < .05). Body weight monitoring (*D*) and macroscopic damage (*E*) of hM4Di water ( n = 9), DSS + Veh ( n = 7), or DSS + CNO ( n = 8) mice. TNF-α (*F*), IL-1β (*G*), and IL-6 (*H*) levels were determined by luminex technology in the colon of hM4Di Water ( n = 4–5), DSS + Veh ( n = 5–6), or DSS + CNO ( n = 6) mice. Statistical analysis was performed using 2-way analysis of variance followed by the Tukey post hoc test (*D*), the Kruskal-Wallis test followed by the Dunn post hoc test (∗∗*P* < .01; ∗∗∗*P* < .001) (*E*, *F*, *H*) or 1-way analysis of variance followed by the Tukey post-hoc test (∗∗*P* < .01) (*G*). Three independent experiments were performed.

The transition from acute to chronic visceral pain is characterized by various adaptations along the neural pain circuits, including microglial activation, a surrogate marker of chronic pathological pain.^11^^,^^15^ Using the DSS model of experimental colitis, we previously found that released microglial granulocyte-colony stimulating factor sensitizes TRPV1^+^ visceral nociceptors that converge onto the spinal dorsal horn.^15^ To determine whether inhibition of TRPV1^+^ visceral afferents could prevent colitis-induced microglial activation and VHS, TRPV1-hM4Di and hM4Di littermate control mice were treated with 2.5% DSS for 7 days and received either saline or chronic CNO treatment (Figure 4*A*). We found that, as previously reported in wild type C57BL/6J mice, TRPV1-hM4Di colitis mice exhibited VHS at 45 and 60 mmHg distension pressures compared with control (Figure 4*B*, 4*C*). Chronic CNO treatment normalized the VHS in TRPV1-hM4Di colitis mice (Figure 4*B*, 4*C*). Notably, the anti-nociceptive effect of CNO correlated with a reduction in neuronal activation in the spinal dorsal horn receiving TRPV1^+^ C-fibers. Following colorectal distension, the number of c-Fos-positive neurons was significantly enhanced in lumbosacral (L6–S1) spinal cord, where axons of the pelvic nerve project^32^ (Figure 4*D*, 4*E*), whereas chemogenetic inhibition of TRPV1^+^ neurons reduced the activation of dorsal horn neurons in L6–S1 spinal cord sections (Figure 4*D*, 4*E*). Importantly, CNO administration in hM4Di littermate control mice (absence of Cre recombinase) (Figure 5*A*) reduced neither DSS-induced VHS (Figure 5*B*) nor the numbers of c-Fos-expressing neurons in the spinal cord (Figure 5*C*), indicating that CNO did not promote a nonspecific analgesic effect that was not hM4Di-mediated.

**Figure 6:**
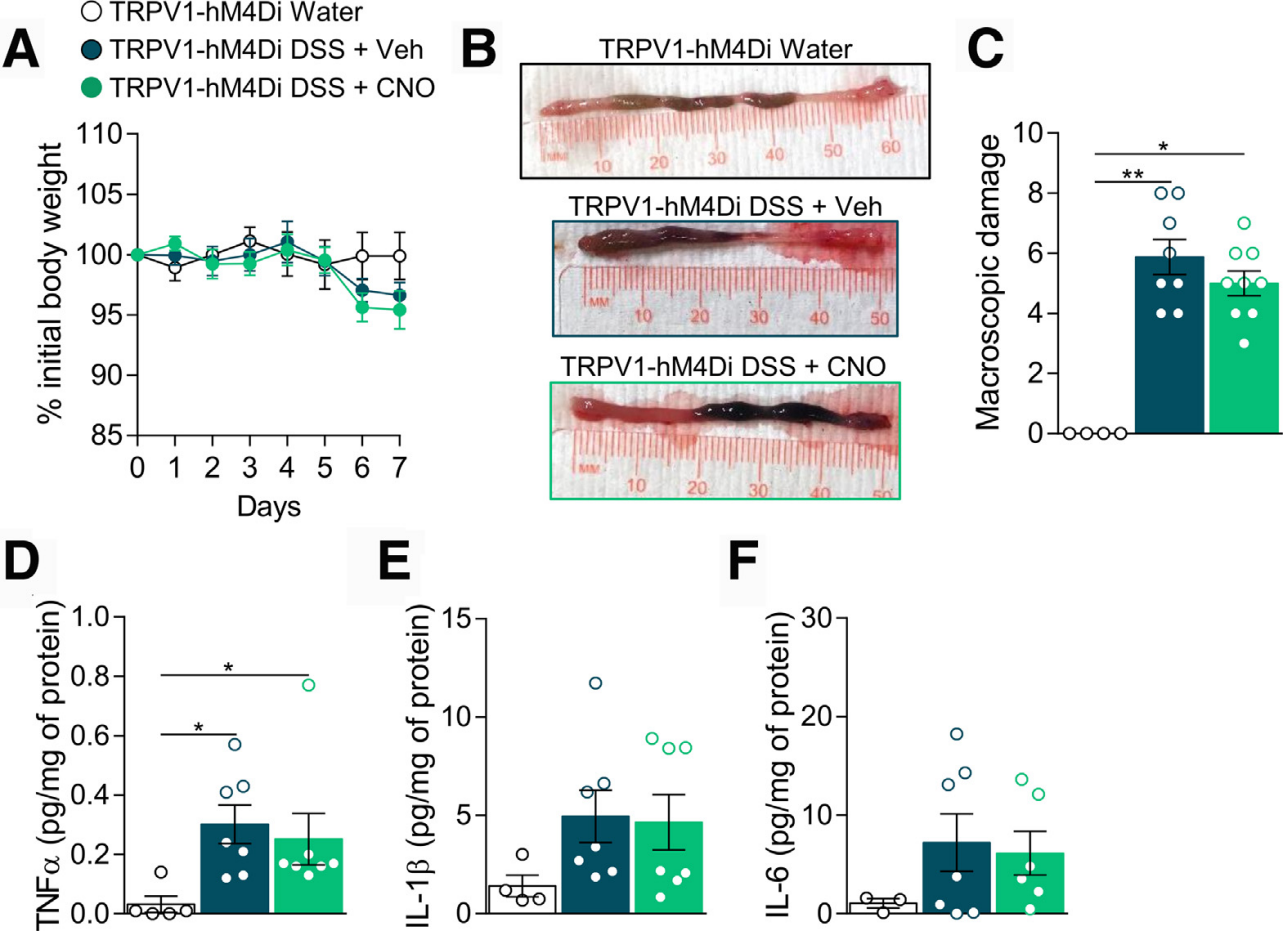
**Chemogenetic silencing of TRPV1**^**+**^**neurons is not sufficient to reduce DSS-induced colitis.** Body weight monitoring (*A*), representative images of colon (*B*), and macroscopic damage (*C*) of TRPV1-hM4Di water ( n = 4), DSS + Veh ( n = 8), and DSS + CNO ( n = 9) mice. TNF-α (*D*), IL-1β (*E*), and IL-6 (*F*) levels were determined by luminex technology in the colon of TRPV1-hM4Di water ( n = 3–5), DSS + Veh ( n = 7), and DSS + CNO ( n = 6–7) treated mice. Statistical analysis was performed using 2-way analysis of variance followed by the Tukey post hoc test (*A*), the Kruskal-Wallis test followed by the Dunn post hoc test (∗*P* < .05; ∗∗*P* < .01) (*B*, *D*, *E*), or 1-way analysis of variance followed by the Tukey post hoc test (*F*). Three independent experiments were performed.

Over the course of 7 days, DSS-treated TRPV1-hM4Di mice developed intestinal inflammation characterized by a slight decrease in the body weight (Figure 6*A*), increase in macroscopic damages (Figure 6*B*, 6*C*) and production of pro-inflammatory cytokines TNF-α, IL1-β, and IL-6 (Figure 6*D* to 6*F*). Production of colonic IL-2, IL-4, IL-10, Il-12p70, monocyte chemoattractant protein-1, and interferon (IFN)-γ was unchanged (Table 1). Chronic CNO treatment did not affect colitis-induced body weight loss, macroscopic damages, cytokine production in TRPV1-hM4Di (Figure 6*A* to 6*F*), or hM4Di littermate controls (Figure 5*D* to 5*H*; Table 1), indicating that inhibition of TRPV1 visceral afferents was not sufficient to reduce the severity of colitis.

**Table 1:** List of the Cytokines Assessed in Colonic Samples of TRPV1-hM4Di and hM4Di Littermate Control Mice

	Cytokine	Water, pg·mg·prot−1 ± SEM	DSS Veh, pg·mg·prot−1 ± SEM	DSS CNO, pg·mg·prot−1 ± SEM	*P* value
TRPV1-hM4Di	IL-2	0.32 ± 0.04899	0.1914 ± 0.03949	0.2414 ± 0.0528	.2186
	IL-4	ND	ND	ND	ND
	IL-10	0.085 ± 0.02661	0.18 ± 0.055598	0.188 ± 0.07618	.4769
	IL-12p70	ND	ND	ND	ND
	MCP-1	15.07 ± 8.82	57.29 ± 13.55	83.19 ± 22.24	.0794
	IFN-γ	ND	ND	ND	ND
hM4Di	IL-2	0.462 ± 0.1435	0.36 ± 0.1362	0.1867 ± 0.02883	.2527
	IL-4	ND	ND	ND	ND
	IL-10	0.1325 ± 0.03924	0.2067 ± 0.1032	0.128 ± 0.05598	.9423
	IL-12p70	ND	ND	ND	ND
	MCP-1	33.12 ± 10.44	47.24 ± 22.42	60.9 ± 18.71	.5788
	IFN-γ	ND	ND	ND	ND

Note: Analysis of multiple cytokines in colon samples from TRPV1-hM4Di or hM4Di control (Water), DSS + Veh, and DSS + CNO mice.

CNO, Clozapine-N-oxide; DSS, dextran sodium sulfate; IFN-γ, interferon-γ; IL, interleukin; MCP-1, monocyte chemoattractant protein-1; ND, not detectable; SEM, standard error of the mean; TRPV1, transient receptor potential vanilloid member 1; Veh, vehicle.

As activation of spinal microglia is a cellular surrogate of VHS in colitis,^15^ we determined whether inhibition of TRPV1^+^ visceral afferents was sufficient to prevent microglial activation in the spinal cord (Figure 4*F*). Following DSS treatment, Iba-1-staining intensity, indicative of microglial activation, was increased in both thoracolumbar (T10–L1) and L6–S1 spinal cord sections of TRPV1-hM4Di mice and control littermates (Figure 4*G*). Chronic CNO treatment significantly reduced Iba-1 expressing microglia in both segments of the spinal cord of TRPV1-hM4Di colitis mice (Figure 4*G*) confirming that chemogenetic inhibition of TRPV1^+^ visceral afferents during DSS regimen is sufficient to reduce spinal microglia activation associated with VHS in colitis.

### Chemogenetic Activation of TRPV1^+^ Visceral Afferents is Sufficient to Promote Visceral Hypersensitivity and Microglia Reactivity in the Spinal Cord

**Figure 7:**
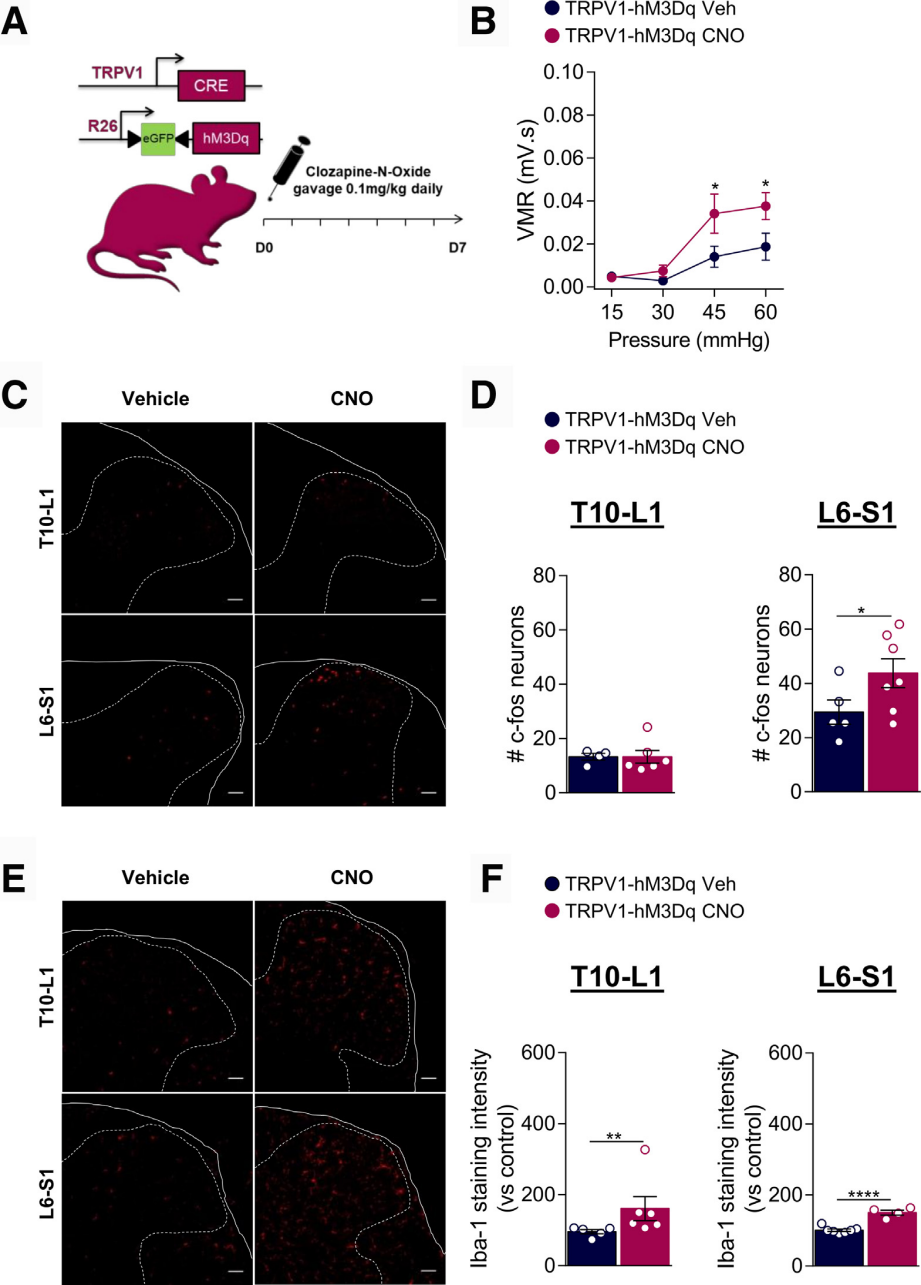
**Activation of TRPV1**^**+**^**visceral afferents induces microglial activation and VHS.***A*, Schematic representation of the experimental design. TRPV1-hM3Dq mice received CNO (0.1 mg/kg) or vehicle, once daily, by gavage for 7 days. *B*, VMR to colorectal distension measured after treatment with Veh ( n = 10) or CNO ( n = 12). Statistical analysis was performed using 2-way analysis of variance followed by the Bonferroni post hoc test (∗*P* < .05). *C*, Representative confocal images of c-Fos positive neurons immunostained in the T10–L1 and L6–S1 spinal cord sections. *D*, Dot plot showing the average number of c-Fos positive neurons in the spinal dorsal horn of Veh ( n = 4–5) and CNO ( n = 6–7) treated mice. Statistical analysis was performed using *t* test (∗*P* < .05). *E*, Representative confocal images of microglia immunoreactive to Iba-1 in the T10–L1 and L6–S1 spinal cord sections. *F*, Dot plot showing the intensity of Iba-1 immunostaining in the spinal dorsal horn of Veh ( n = 5–7) and CNO ( n = 4–6) treated mice. Statistical analysis was performed using the Mann-Whitney or *t* test (∗∗*P* < .01; ∗∗∗∗*P* < .0001). Three independent experiments were performed.

**Figure 8:**
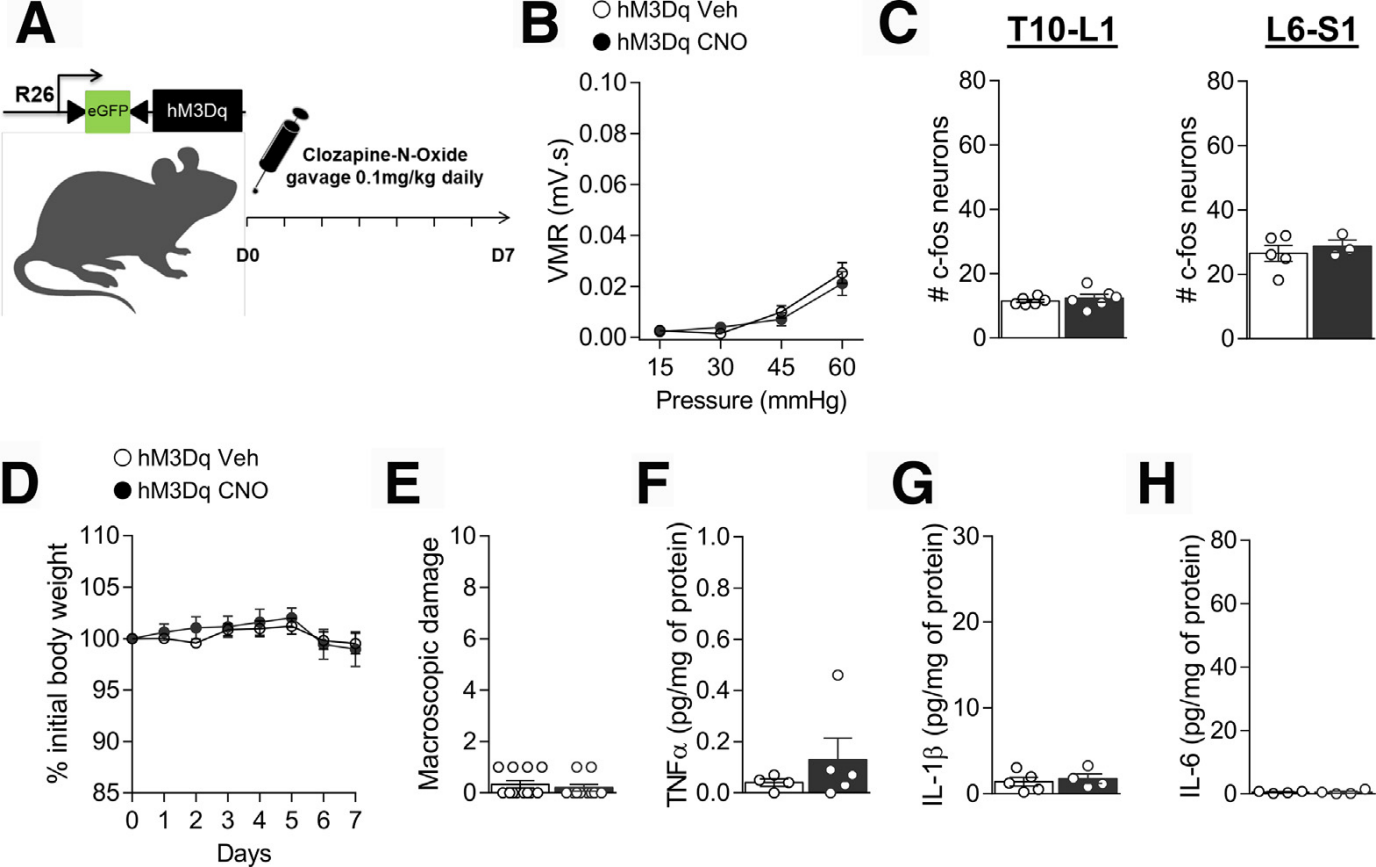
**CNO has no pronociceptive or proinflammatory effect in hM3Dq littermate controls.***A*, Schematic representation of the experimental design. hM3Dq mice received vehicle as control (Veh) or CNO (0.1 mg/kg) by gavage once daily for 7 days. *B*, VMR to colorectal distension after treatment with Veh ( n = 12) or CNO ( n = 10). Statistical analysis was performed using 2-way analysis of variance followed by the Bonferroni post hoc test. *C*, Dot plot showing the average number of c-Fos positive neurons in the spinal dorsal horn of Veh ( n = 5–6) or CNO ( n = 3–6) treated mice. Statistical analysis was performed using the *t* test. Body weight monitoring (*D*) and macroscopic damage (*E*) of hM3Dq Veh ( n = 12) or CNO ( n = 10) treated mice. TNF-α (*F*), IL-1β (*G*), and IL-6 (*H*) levels were determined by luminex technology in the colon of hM3Dq Veh ( n = 4–5) or CNO ( n = 4–5) treated mice. Statistical analysis was performed using 2-way analysis of variance followed by the Bonferroni post hoc test, Mann-Whitney test, or *t* test. Three independent experiments were performed.

As chronic silencing of TRPV1^+^ afferents was able to prevent VHS and microglial activation in the spinal cord, we next investigated whether TRPV1^+^ neurons activation was sufficient to trigger microglial activation associated with VHS in the absence of colitis. To chronically activate TRPV1^+^ visceral afferents, TRPV1-hM3Dq and hM3Dq littermate control mice received vehicle or CNO by gavage once daily for 7 days (Figure 7*A*). Chemogenetic activation of TRPV1^+^ visceral afferents induced VHS in response to mild and noxious colorectal distension, compared with control (Figure 7*B*). Accordingly, activation of spinal cord neurons was significantly enhanced in L6–S1 spinal cord sections of CNO-treated TRPV1-hM3Dq mice (Figure 7*C*, 7*D*). Importantly, CNO did not induce VHS or spinal neuron activation in the absence of the TRPV1-cre allele (hM3Dq mice), thus excluding a pronociceptive role of CNO itself (Figure 8*A* to 8*C*).

**Figure 9:**
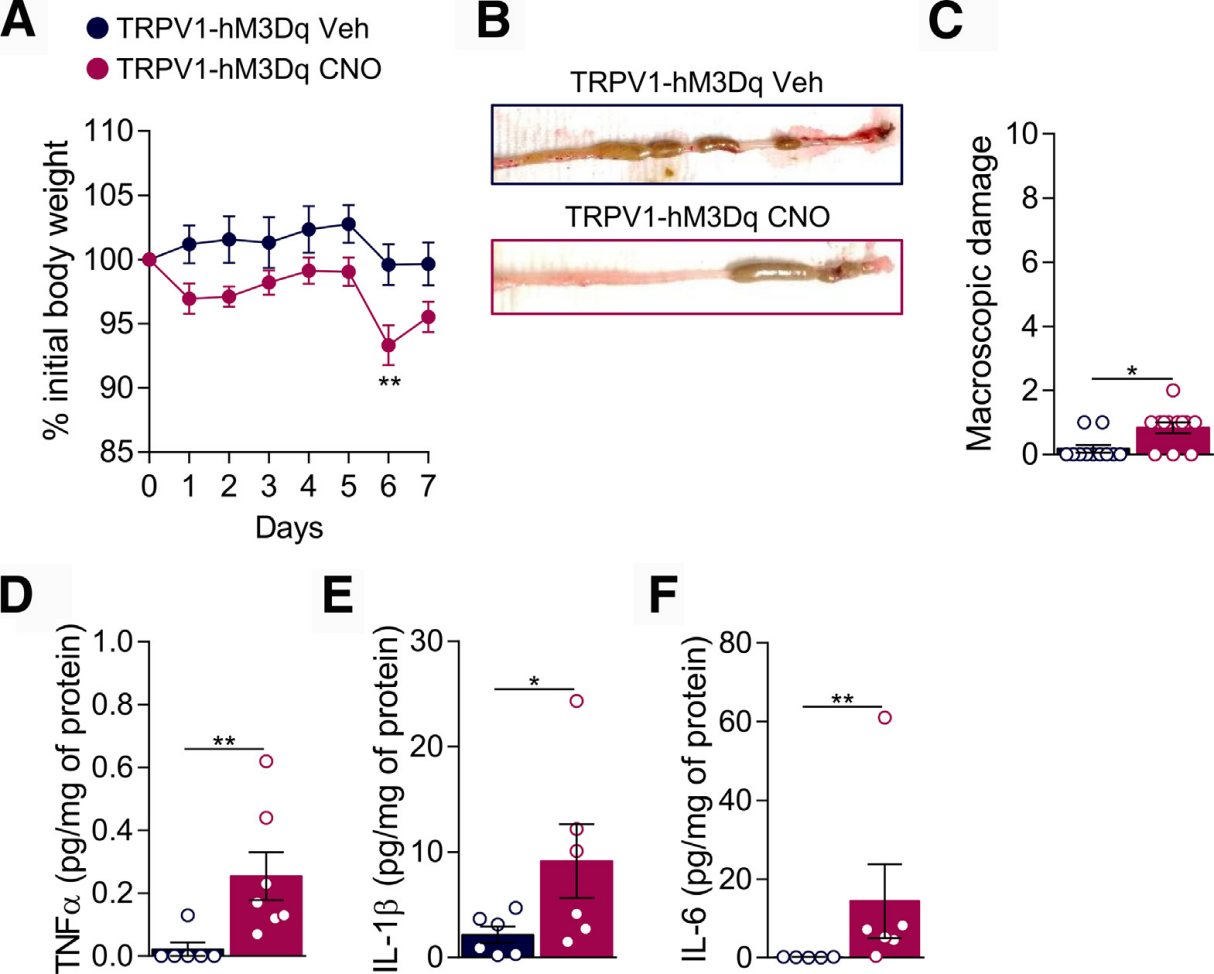
**Chemogenetic activation induces neurogenic inflammation in naive mice.** Body weight monitoring (*A*), representative images of colon (*B*), and macroscopic damage (*C*) of TRPV1-hM3Dq Veh ( n = 11) and CNO ( n = 12) treated mice. TNF-α (*D*), Il-1β (*E*), and IL-6 (*F*) levels were determined by luminex technology in the colon of TRPV1-hM3Dq Veh ( n = 5–6) and CNO ( n = 6–7) treated mice. Statistical analysis was performed using 2-way analysis of variance followed by the Bonferroni post hoc test (∗∗*P* < .01), Mann-Whitney test, or *t* test (∗*P* < .05; ∗∗*P* < .01). Three independent experiments were performed.

Notably, activation of TRPV1^+^ visceral afferents in naïve mice was sufficient to induce an inflammatory response in the colon. Body weight was reduced in CNO-treated TRPV1-hM3Dq mice (Figure 9*A*), and macroscopic damage (Figure 9*B*, 9*C*) and colonic cytokines (TNF-α, IL-1β, and IL-6) were all elevated (Figure 9*D* to 9*F*). Although the monocyte chemoattractant protein-1 colonic level was increased, CNO treatment did not induce the production of colonic IL-2, IL-4, IL-10, Il-12p70 or IFN-γ in TRPV1-hM3Dq mice (Table 2). Furthermore, we did not detect changes in macroscopic parameters and cytokine production in control hM3Dq mice, indicating that the observed inflammatory response was due to the specific CNO-mediated activation of TRPV1^+^ visceral afferents (Figure 8*D* to 8*H*; Table 2).

**Table 2:** List of the Cytokines Assessed in Colonic Samples of TRPV1-hM3Dq and hM3Dq Littermate Control Mice

	Cytokines	Veh, pg·mg·prot−1 ± SEM	CNO, pg·mg·prot−1 ± SEM	*P* value
TRPV1-hM3Dq	IL-2	0.4667 ± 0.1796	0.4663 ± 0.1157	.991
	IL-4	ND	ND	ND
	IL-10	0.1633 ± 0.04897	0.1233 ± 0.04169	.5479
	IL-12p70	ND	ND	ND
	MCP-1	4.565 ± 1.488	98.38 ± 38.7	.0180^a^
	IFN-γ	ND	ND	ND
hM3Dq	IL-2	0.4975 ± 0.1743	0.266 ± 0.03473	.1905
	IL-4	ND	ND	ND
	IL-10	0.348 ± 0.1316	0.065 ± 0.03329	.105
	IL-12p70	ND	ND	ND
	MCP-1	12.31 ± 3.757	16.37 ± 1316	.3095
	IFN-γ	ND	ND	ND

Note: Analysis of multiple cytokines in colon samples from TRPV1-hM3Dq or hM3Dq control (Veh), and CNO mice.

CNO, Clozapine-N-oxide; IFN-γ, interferon-γ; IL, interleukin; MCP-1, monocyte chemoattractant protein-1; ND, not detectable; SEM, standard error of the mean; TRPV1, transient receptor potential vanilloid member 1; Veh, vehicle.

a *t* test (*P* < .05)

We next examined Iba-1 staining intensity in the spinal dorsal horn following CNO treatment. Chronic stimulation of TRPV1^+^ visceral afferents enhanced Iba-1 immunoreactivity in both T10–L1 and L6–S1 spinal cord sections as found in DSS condition (Figure 7E, 7*F*).

### TRPV1^+^ Visceral Afferents Activate Microglia Through ATP/P2RY12 Signaling

**Figure 10:**
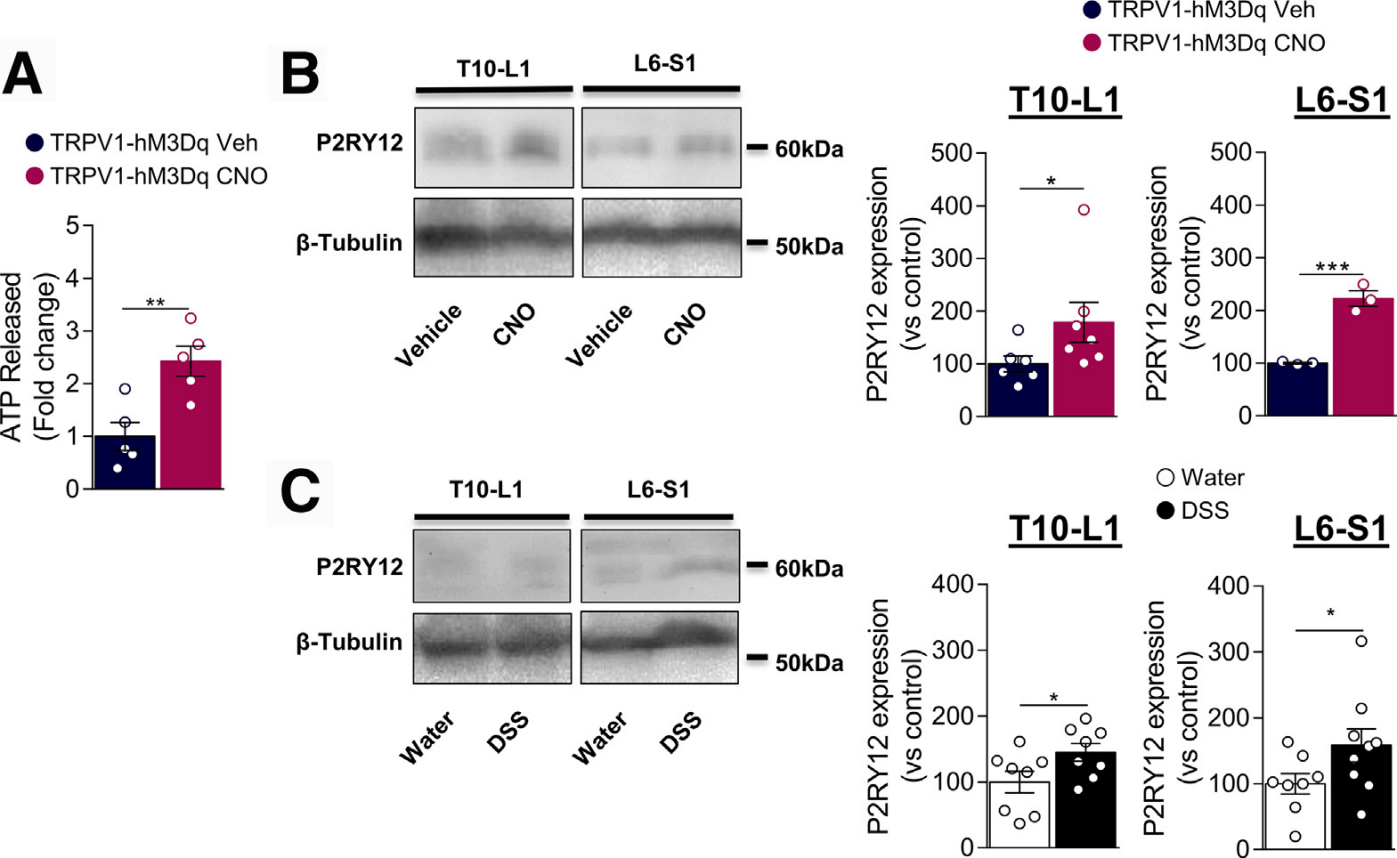
**Activation of TRPV1**^**+**^**neurons induces ATP release and P2RY12 upregulation in the spinal cord.***A*, ATP levels in supernatant of control (Veh,  n = 5) or CNO-treated (10 μM,  n = 5) TRPV1-hM3Dq neurons. Data are normalized to control treated neurons. Statistical analysis was performed using the *t* test (∗∗*P* < .01). *B*, Purinergic receptor P2RY12 protein level was determined by Western blot in the T10–L1 and L6–S1 spinal cord sections of Veh ( n = 3–6) and CNO-treated ( n = 3–7) TRPV1-hM3Dq mice. *C*, Bar graph representing P2RY12 quantification in T10–L1 and L6–S1 spinal cord sections. Data are normalized to Veh mice. Statistical analysis was performed using the Mann-Whitney or *t* test (∗*P* < 0.05; ∗∗∗*P* < .001). *D*, Purinergic receptor P2RY12 protein level was determined by Western blot in the T10–L1 and L6–S1 spinal cord sections of control ( n = 8) and DSS ( n = 8–9) wild type mice. *E*, Bar graph representing P2RY12 quantification in T10–L1 and L6–S1 spinal cord sections. Data are normalized to control mice. Statistical analysis was performed using the *t* test (∗*P* < .05; ∗∗∗*P* < .001). Two independent experiments were performed.

The release of adenosine triphosphate (ATP) stored in vesicles from nerve terminals is an important mechanism of sensory neurotransmission in the spinal cord.^33^ Extracellular ATP acts as an excitatory neurotransmitter in the central nervous system (CNS), and microglia respond to ATP through purinergic receptors, regulating microgliosis and cell motility.^34^ To determine whether chemogenetic activation of TRPV1^+^ neurons was sufficient to mediate ATP release, isolated DRG neurons from TRPV1-hM3Dq mice were stimulated with CNO, and extracellular ATP was measured in the supernatant. As shown in Figure 10*A*, acute activation of TRPV1-hM3Dq neurons induced a 2-fold increase of ATP release. Growing evidence suggests the importance of microglial purinergic receptors in chronic pain states. Among them, P2RY12 is a specific marker of microglia^35^ implicated in neuropathic and inflammatory pain.^9^^,^^36^ Thus, we evaluated P2RY12 expression in the spinal cord of CNO-treated TRPV1-hM3Dq mice. Seven days after CNO treatment, P2RY12 protein level was increased in both T10–L1 and L6–S1 spinal cord sections from TRPV1-hM3Dq mice compared with the vehicle group (Figure 10*B*). Importantly, P2RY12 was also upregulated in the spinal cord of DSS-treated mice, suggesting a role of spinal microglial P2RY12 in colitis-induced VHS (Figure 10*C*).

### P2RY12 Promotes Microglial Activation in Colitis

**Figure 11:**
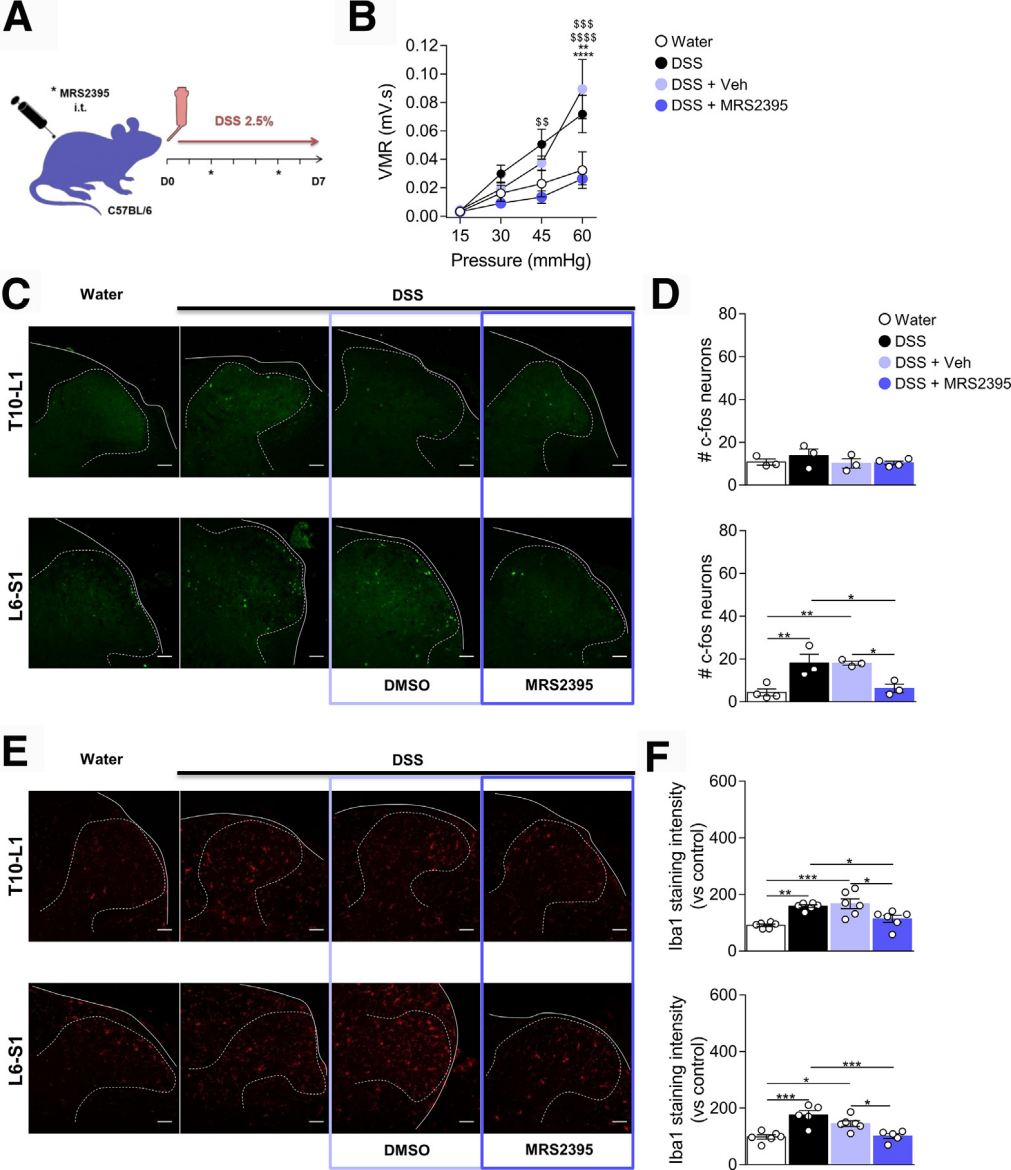
**P2RY12 antagonist blocks colitis-induced microglial activation and VHS.***A*, Schematic representation of the experimental design. Mice treated with DSS for 7 days received 2 intrathecal injections of MRS2395 or vehicle at D2 and D5. *B*, VMR to colorectal distension after treatment with Water ( n = 6), DSS ( n = 6), DSS + Vehicle ( n = 7) or DSS + MRS2395 ( n = 8). Statistical analysis was performed using 2-way analysis of variance followed by the Tukey post hoc test (∗∗*P* < .01; ∗∗∗*P* < .001 vs Water; ^$$^*P* < .01; ^$$$^*P* < .001; ^$$$$^*P* < .0001 vs DSS + MRS2395). *C*, Representative confocal images of c-Fos positive neurons the T10–L1 and L6–S1 spinal cord sections. Scale bar = 50 μm. *D*, Dot plot showing the average number of c-Fos positive neurons in the spinal dorsal horn of Water ( n = 3–4), DSS ( n = 3), DSS + vehicle ( n = 3), or DSS + MRS2395 ( n = 3–4) treated mice. Statistical analysis was performed using 1-way analysis of variance followed by the Tukey post hoc test (∗*P* < .05; ∗∗*P* < .01). *E*, Representative confocal image of microglia immunoreactive to Iba-1 in the T10–L1 and L6–S1 spinal cord sections. *F*, Dot plot showing the intensity of Iba-1 immunostaining in the spinal dorsal horn of Water ( n = 6), DSS ( n = 5–6), DSS + vehicle ( n = 6), or DSS + MRS2395 ( n = 5–6) treated mice. Statistical analysis was performed using 1-way analysis of variance followed by the Tukey post hoc test (∗*P* < .05; ∗∗*P* < .01; ∗∗∗*P* < .001). Three independent experiments were performed.

To test the role of P2RY12 in colitis-induced microglial activation, mice were treated with the P2RY12 blocker MRS2395^9^ or vehicle control during DSS regimen (Figure 11*A*). Although DSS-treated mice (both saline- and vehicle-injected groups) exhibited a significant increase in visceromotor response (VMR) to colorectal distension (CRD), pharmacological inhibition of spinal P2RY12 by MRS2395 completely blocked colitis-induced VHS compared with the vehicle-injected group (Figure 11*B*). Accordingly, blockade of microglial P2RY12 reduced spinal cord neuron activation in the L6-S1 spinal cord sections of colitis mice (Figure 11*C*, 11*D*).

**Figure 12:**
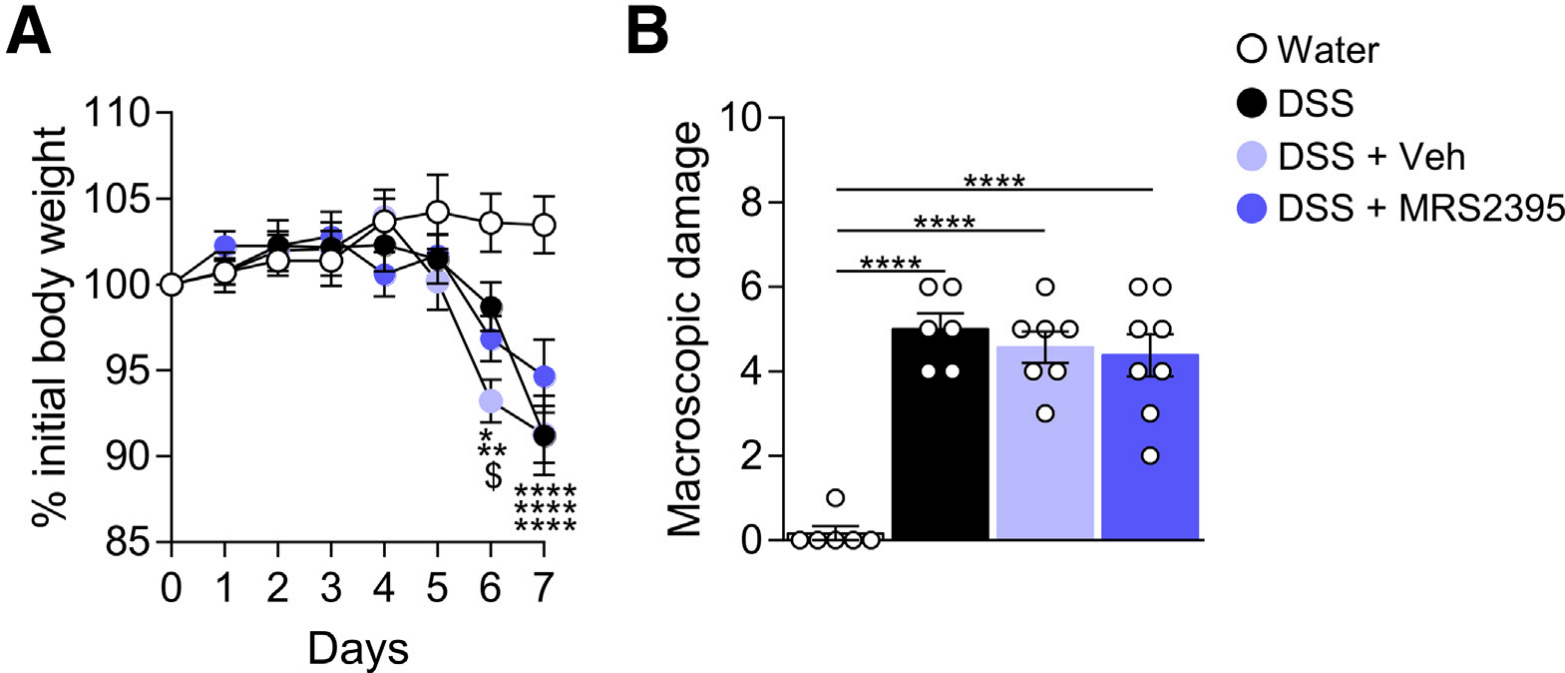
**P2RY12 antagonist has no effect on the severity of DSS-induced colitis.** Body weight monitoring (*A*) and macroscopic damage (*B*) of Water ( n = 6), DSS ( n = 6), DSS + Vehicle ( n = 7), or DSS + MRS2395 ( n = 8) mice. Statistical analysis was performed using 2-way analysis of variance followed by the Tukey post hoc test (∗*P* < .05; ∗∗*P* < .01; ∗∗∗∗*P* < .0001 vs Water; ^$^*P* < .05 vs DSS + Veh) or the Kruskal-Wallis test followed by the Dunn post hoc test (∗∗*P* < .01). Three independent experiments were performed.

As P2RY12 antagonists are widely used as antiplatelet medication,^37^ we assessed the severity of DSS-induced inflammation after MRS2395 administration. DSS-treated mice exposed to MRS2395 compound lost the same percentage of their initial body weight as compared with respective controls (Figure 12*A*). Evaluation of the macroscopic damage did not show any differences between mice receiving vehicle or MRS2395 (Figure 12*B*). Altogether, these data indicate that inhibition of spinal P2RY12 with MRS2395 alleviates DSS-induced VHS without affecting severity of colitis.

We next examined whether P2RY12 inhibition prevented microglial activation in the spinal cord during colitis. Although Iba-1 immunoreactivity was increased in both T10–L1 and L6–S1 spinal cord sections of colitis mice (Figure 11*E*, 11*F*), administration of MRS2395 at days 2 and 5 of DSS reduced Iba-1 expressing microglia in both segments of the spinal cord (Figure 11*E*, 11*F*). These data confirm that activation of P2RY12 triggers microglia reactivity in the spinal cord of colitis mice.

### Blockade of Spinal P2RY12 Prevents Post-colitis Persistent Visceral Hypersensitivity

**Figure 13:**
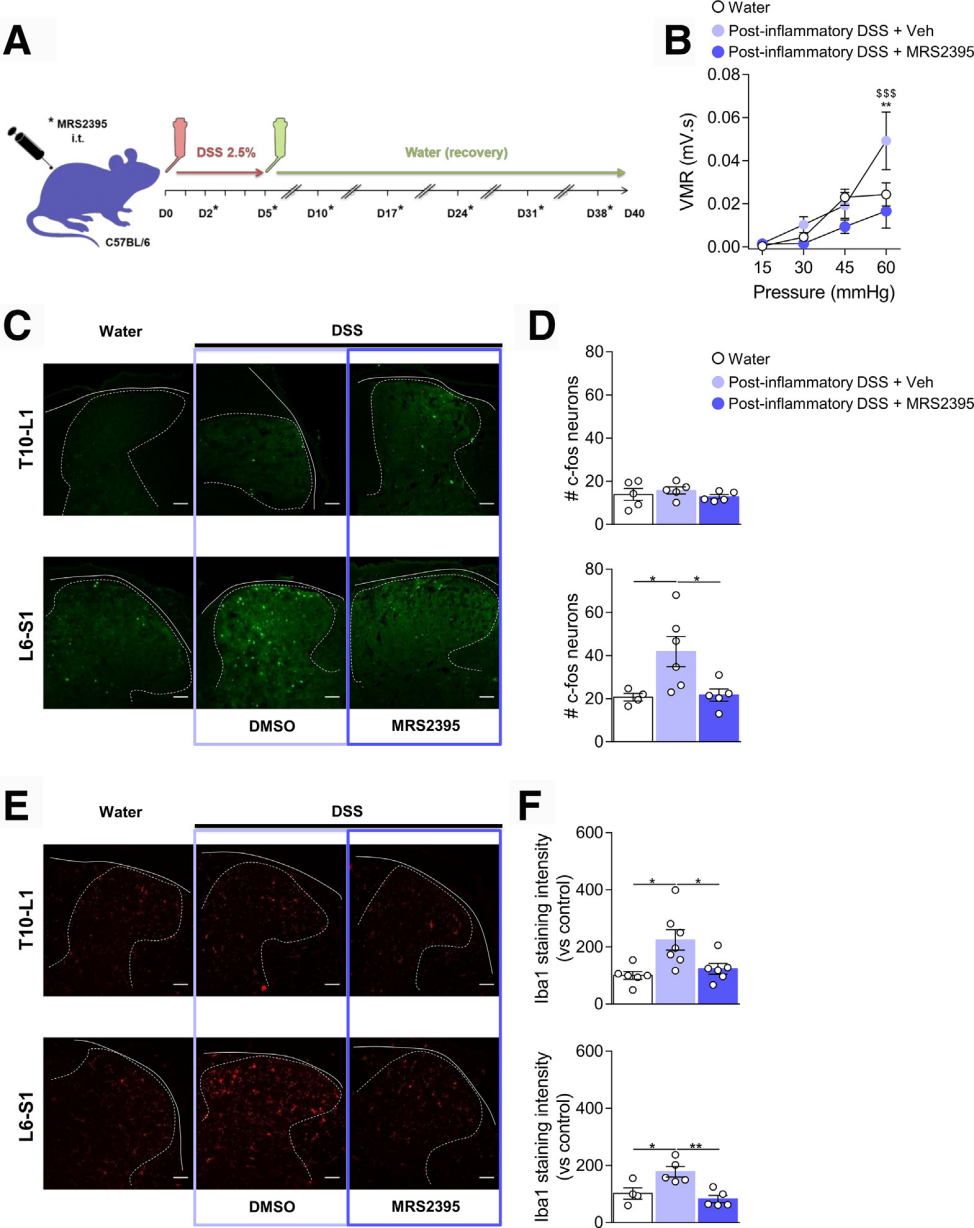
**Blocking spinal P2RY12 prevents visceral hypersensitivity during the remission phase of colitis.***A*, Schematic representation of the experimental design. Colitis was induced by adding 2.5% DSS in the drinking water for 5 days after which mice received only water for the next 5 weeks. Mice were treated at D2 and D5 during DSS exposure then once a week during the recovery period with either MRS2395 or vehicle control. *B*, VMR to colorectal distension after treatment with Water ( n = 8), post-inflammatory DSS + Vehicle ( n = 9), or post-inflammatory DSS + MRS2395 ( n = 9). Statistical analysis was performed using 2-way analysis of variance followed by the Tukey post hoc test (∗∗*P* < .01 vs Water; ^$$$^*P* < .001 vs DSS + MRS2395). *C*, Representative confocal images of c-Fos positive neurons in T10–L1 and L6–S1 spinal cord sections. Scale bar = 50 μm. *D*, Dot plot showing the average number of c-Fos positive neurons in the spinal dorsal horn of Water ( n = 4–5), post-inflammatory DSS + vehicle ( n = 5), or post-inflammatory DSS + MRS2395 ( n = 5) treated mice. Statistical analysis was performed using 1-way analysis of variance followed by the Tukey post hoc test (∗*P* < .05). *E*, Representative confocal image of microglia immunoreactive to Iba-1 in the T10–L1 and L6–S1 spinal cord sections. *F*, Dot plot showing the intensity of Iba-1 immunostaining in the spinal dorsal horn of Water ( n = 4–6), post-inflammatory DSS + vehicle ( n = 5–7), or post-inflammatory DSS + MRS2395 ( n = 5–6) treated mice. Statistical analysis was performed using 1-way analysis of variance followed by the Tukey post-hoc test (∗*P* < .05; ∗∗*P* < .01). Two independent experiments were performed.

**Figure 14:**
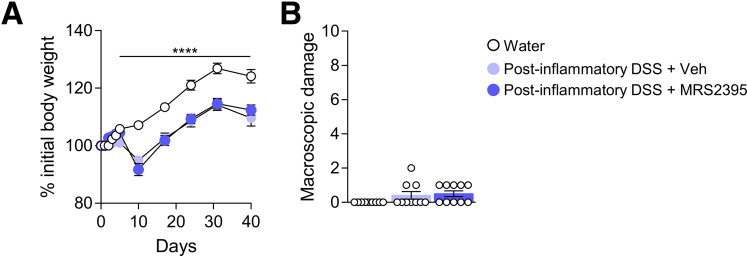
**P2RY12 antagonist has no effect on the resolution of inflammation post colitis.** Body weight monitoring (*A*) and macroscopic damage (*B*) of Water ( n = 9), Post-inflammatory DSS + Vehicle ( n = 10), or Post-inflammatory DSS + MRS2395 ( n = 10) mice. Statistical analysis was performed using 2-way analysis of variance followed by the Tukey post hoc test (∗∗∗∗*P* < .0001 vs Water) or the Kruskal-Wallis test followed by the Dunn post hoc test. Two independent experiments were performed.

As pharmacological inhibition of P2RY12 was able to prevent both microglial activation and VHS in acute colitis, we hypothesized that P2RY12 activation could prime spinal microglia leading to chronic VHS during the recovery phase of colitis. We thus tested the effect of MRS2395 using a previously characterized model of post-inflammatory VHS.^18^ Briefly, mice were given 2.5% DSS for 5 days, after which they received MRS2395 (or vehicle control) once a week for 5 weeks during the recovery period (Figure 13*A*). As previously reported, post-colitis mice exhibited a significant increase in VMR to CRD (Figure 13*B***)**, confirming VHS despite resolution of inflammation. Mice were back to their original weight 1 week post-DSS discontinuation (Figure 14*A*) and showed no significant changes in macroscopic damage (Figure 14*B*). Importantly, blocking of P2RY12 did not affect the recovery curve of DSS-treated mice (Figure 14*A*, 14*B*). As found with the acute DSS model, inhibition of spinal P2RY12 normalized VHS in post-colitis mice (Figure 13*B*). These results correlated with a reduction in neuronal activation in the L6–S1 spinal cord sections of MRS2395 treated mice (Figure 13*C*, 13*D*).

We next evaluated microglia activation in the post-colitis model. Iba-1 immunoreactivity was increased in both T10–L1 and L6–S1 spinal cord sections of DSS mice in the post-inflammatory phase (Figure 13*E*, 13*F*). Administration of the P2RY12 antagonists during the acute and recovery phases reduced Iba-1 expressing microglia in both segments of the spinal cord, indicating a pivotal role of spinal P2RY12 activation in priming central sensitization induced by acute flares and remissions.

## Discussion

Central sensitization plays an integral role in maintaining pain even when the tissue or injury has healed.^38^ This process, occurring at spinal and supraspinal levels, changes the responsiveness of visceral afferents and amplify painful signals in the gastrointestinal tract.^39^ Accumulating evidence suggests that central sensitization is also driven by neuroinflammation through glial cells activation in the spinal cord.^40^ In the present study, we showed that TRPV1-expressing visceral afferents control microglial reactivity in colitis-induced VHS. We demonstrated that chemogenetic inhibition of TRPV1^+^ neurons prevents microglial activation associated with VHS in experimental colitis. Furthermore, activation of TRPV1^+^ visceral afferents, in the absence of disease, is sufficient to drive both VHS and microglial reactivity in the spinal cord. We next showed that hM3Dq-expressing TRPV1^+^ neurons release ATP in an activity-dependent manner. This leads to an upregulation of the microglial marker P2RY12, concomitant with an activation of microglia. Accordingly, intrathecal injection of the selective P2RY12 antagonist, MRS2395, prevented microglial activation and VHS, suggesting that ATP-P2RY12 signaling in the microglia drives central sensitization underlying chronic visceral pain. Finally, we showed that chronic treatment with the P2RY12 antagonist alleviates sustained VHS post-colitis. Together, our results suggest that communication between ATP-releasing TRPV1^+^ visceral afferents and P2RY12 signaling in the spinal cord microglia orchestrates the establishment of VHS that persists in remission.

DREADD receptors have been extensively used to silence or enhance neuronal firing in the CNS and modulate cellular signaling through Gi, Gq, Gs, or β-arrestin cascades.^22^ Unlike optogenetic that targets a minimum range of tissue with the arc light of an optic fiber, CNO application in DREADD-expressing animals penetrate a wider mucosal surface and receptive fields of the colon, providing a tonic activation of the neurons. Furthermore, activation of DREADDs affects neuronal activity in a time-dependent fashion,^23^ while preserving neuronal function and thus reducing off target effects that can be caused by chemical ablation or denervation.^41^^,^^42^ As previously reported,^16^ a small subset of nonpeptidergic neurons was found to express the DREADDs receptors, likely due to a wider range of DRG neurons that transiently express TRPV1 during development.^25^ However, we did not notice DREADD expression in non-neuronal cells centrally or peripherally, and administration of CNO to DREADD littermate controls did not impact colon inflammation or VHS, indicating that microglial and behavioral responses to CNO were both neuron-mediated.

Nociceptors, which have cell bodies in DRGs, transmit noxious stimuli from the gut to the spinal cord. Besides their anatomical properties, nociceptors have been classified based on channel and receptor expression. Recently, colon-innervating DRG neurons have been categorized into 7 classes among which 6 express the TRPV1 channel.^16^ Initially described as the receptor for capsaicin, TRPV1 responds to a variety of stimuli including lipids and low pH in the inflamed colon.^17^ Furthermore, growing evidence suggests that sensitization of TRPV1 by receptors of inflammatory mediators, such as mastocyte-derived histamine, mediates VHS.^19^^,^^20^ Strikingly, despite the importance of visceral sensitization in IBD, the first line of drugs for pain management in IBD remains nonsteroidal anti-inflammatory drugs, opiates, and antidepressants.^43^ This is in part due to a limited understanding of the neural pain circuits of visceral pain and the neuro-immune processes that promote pain sensitization in the spinal cord. Although hyperexcitability and neuroplastic changes of visceral afferents is a major component of VHS, direct or indirect mechanisms of sensitization, through macrophages, dendritic cells, or enterocytes, have been suggested.^44^^,^^45^ Here, using chemogenetics, we hypothesized that TRPV1^+^ neuron activity is solely responsible for initiating and establishing VHS. Given that spinal microglia are indispensable for pain chronicity in mice,^40^ our findings support a model wherein microglia sense and respond to neuronal activity in TRPV1^+^ visceral afferents that project to the spinal dorsal horn, and interfering with hyperexcitable TRPV1^+^ visceral neurons is sufficient to prevent microglial activation underlying VHS. We noticed a discrepancy in the number of c-Fos-positive neurons between the T10–L1 and L6–S1 spinal cord sections. As previously described, visceral afferents in splanchnic and pelvic pathways differentially contribute to the recruitment of spinal pathways following colorectal distension.^46^^,^^47^ Under colonic inflammation, repetitive noxious CRD (80 mmHg; 30 seconds on; 90 seconds off for 2 hours) enhanced c-Fos activation in the T10–L1 spinal cord.^48^ However, we did not apply repetitive noxious distensions, which may explain the absence of c-Fos activation in the T10–L1 spinal sections. Also, the sensitization of peptidergic neurons is necessary, but not sufficient, to induce c-Fos in the spinal cord,^49^ suggesting that the combination of inflammation and CRD induce c-Fos upregulation in the L6–S1 but not in the T10–L1 spinal sections. Overall, our work provides a causal relationship between TRPV1^+^ visceral afferent activity, microglial response, and VHS in colitis.

Microglial activation has been well-described in a range of neurological disorders, including chronic pain. In addition to their role as sentinels of the CNS, microglia play a central role in neurodevelopment and in the maintenance of neuronal homeostasis, sensing neuronal activity driven by sensory input and damage of peripheral nerves and the spinal cord.^50^ In preclinical models of colitis, intestinal inflammation induced by 2,4,6-trinitrobenzene sulfonic acid, dinitrobenzene sulfonic acid, or DSS correlate with microglial reactivity in the spinal cord, and subsequent VHS.^11^^,^^14^^,^^15^ Ablating microglia or preventing their activation in vivo alleviates VHS.^11^^,^^15^ TRPV1^+^ C-fibers were previously reported to drive microglial activation in preclinical models of peripheral inflammation and neuropathic pain.^51^ Furthermore, intraplantar capsaicin alone mediates upregulation of Iba-1-positive microglia in the spinal cord,^51^ and sciatic nerve stimulation of C-fibers elicits prolonged microglial-induced central sensitization in the absence of injury.^52^ We thus hypothesized that a direct crosstalk between TRPV1^+^ visceral afferents and microglia may be a key process of central sensitization in persistent visceral pain. Our results indicate that chemogenetic activation of TRPV1^+^ visceral neurons, in the absence of colitis, is sufficient to induce microglia reactivity and VHS. Collectively, our data provide evidence of a direct TRPV1^+^ neuron-microglia interaction in colitis pain and suggest that restraining P2RY12 signaling in microglia, via chronic intervention on TRPV1^+^ neurons, will prevent VHS, despite colitis.

TRPV1^+^ neurons are peptidergic C-fibers, for the most past, based on their expression of neuropeptides such as substance P, CGRP, somatostatin, neuromedin, neurotensin, and natriuretic polypeptide b.^16^ The release of neuropeptides at the periphery are pro- or anti-inflammatory depending on the pathological context and the tissue innervated.^53^, ^54^, ^55^ Recently, Chen and colleagues showed that optogenetic activation of cutaneous TRPV1^+^ neurons drives a Th17 response.^56^ Our results suggest that chemogenetic inhibition of TRPV1^+^ neurons is not sufficient to reduce DSS-induced colitis, whereas CNO stimulation induces neurogenic inflammation in the colon, highlighted by TNF-α, IL-1β, or IL-6 upregulation. A chemogenetic intervention on TRPV1^+^ neurons, unlike chemical ablation by capsaicin or surgical denervation, is reversible and lasts for only a few hours,^23^ which may explain the lack of efficacy of TRPV1 neuron silencing on mucosal inflammation. We also acknowledge that there is some heterogeneity in the severity of colitis between mice under DSS regimen (Figure 6). This may be due to environmental factors, including the microbiota composition and an imbalance of the mucosal immune system, which play critical roles in the development of DSS-induced colitis.^57^ TNF-α and IFN-γ are key cytokines in IBD,^58^ and although TNF-α production was significantly increased in our experiments, IFN-γ was not detected. As IFN-γ is mainly produced by CD4+ Th1 cells^59^^,^^60^ that are activated at a later phase of DSS colitis (day 10),^61^, ^62^, ^63^ it is possible that the number of memory T cells in the colonic mucosa is insufficient to measure IFN-γ at 7 days of DSS regimen.^64^ However, the DSS model involves both innate and adaptive responses associated with visceral hypersensitivity that have been described in IBD.^58^

Several mediators, including chemokines (CX3CL1, CCL2, CXCL13), neuropeptides (substance P and CGRP), and colony-stimulating factor 1, have been involved in microglial activation.^65^ Among these, ATP plays a central role in targeting microglial purinergic receptors.^34^ Although it is usually assumed that damaged neurons release ATP, growing evidence shows that neuronal activity also contributes to ATP release in the spinal cord.^66^^,^^67^ The source of ATP remains unclear, but some evidence highlights the role of nociceptors, as TRPV1 activation by capsaicin enhances ATP in the spinal cord.^68^ Using DREADD, we observed an increase in ATP release following selective activation of cultured TRPV1^+^ neurons by CNO. Monitoring ATP elevation in a living animal would strongly support our model and help to better understand the cascade of events initiating and maintaining visceral hypersensitivity. Such approach could be done using available sensitive and selective biosensors expressed in microglial cells of the spinal cord.^69^, ^70^, ^71^ Microglia express several subtypes of ionotropic (P2XRs) and metabotropic purinergic receptors (P2YRs), which detect synaptic release of ATP.^34^ The nature of purinergic responses is dose-dependent; low concentrations of ATP act via P2YRs, but high concentrations (millimolar range) activate P2XRs.^72^^,^^73^ Although synaptic ATP level of activated neurons can reach micromolar concentration range,^67^ ATP is rapidly hydrolyzed by ecto-nucleotidases activating P2YRs.^74^ Among them, the P2RY12 is an inhibitory G protein-coupled receptor activated by adenosine diphosphate, reducing adenylyl cyclase.^75^ Highly expressed in platelets, its expression is restricted to microglia in the CNS^35^^,^^76^ and controls ATP/adenosine diphosphate-dependent chemotaxis and motility of microglia.^77^ Under disease conditions, the expression of P2RY12 at both the mRNA and protein level is upregulated, promoting pathological pain.^8^^,^^78^^,^^79^ Here, we report that selective activation of TRPV1^+^ visceral neurons is sufficient to drive P2RY12 expression in the spinal cord, as found in DSS colitis. Our data highlight P2RY12 as a surrogate marker of microglia activation associated with colitis-induced VHS.

The role of P2RY12 in microglial activation has been reported in various preclinical models of persistent pain. Selective P2RY12 antagonists attenuate microglia activation, mechanical allodynia, and thermal hyperalgesia in models of neuropathic, inflammatory, and cancer pain.^8^^,^^9^^,^^36^^,^^79^ Furthermore, P2RY12-deficient mice experience less pain in a neuropathic pain model.^8^ P2RY12 antagonists (eg, clopidogrel bisulfate) are widely prescribed as anti-platelet drugs for reducing rapid thrombocyte aggregation.^37^^,^^80^ Therefore, we decided to restrict P2RY12 antagonist administration to the spinal cord, and we used a selective antagonist of P2RY12 receptors, MRS2395, that did not affect the severity of colitis. When considering P2RY12 antagonism for pain management in IBD and IBS, it will be important to ensure that receptor inhibition is restricted to the CNS.

As a relapsing and remitting condition, IBD is often associated with debilitating abdominal pain, which can persist despite endoscopic remission and is reported as IBS-like symptoms.^4^^,^^81^^,^^82^ Colonic inflammation can cause subsequent sensitization of visceral afferents that persists long after the resolution of inflammation.^14^^,^^18^ This chronic disorder of the gut-brain axis is caused by both peripheral and central mechanisms^44^ that drive visceral hypersensitivity in the post-inflammatory model used in our study (Figures 13 and 14). Although microglial activation persists post DSS colitis,^14^ we showed that inhibiting microglial P2RY12 during both the acute and recovery phase of colitis could maximize the reduction of VHS, thus supporting analgesic strategies that target P2RY12 chronically.

Overall, our work shows that TRPV1^+^ nociceptor-microglia interactions can precipitate the establishment of persistent visceral pain following colitis, through a central mechanism involving microglial P2RY12. Thus, purinergic signaling in the spinal cord could be harnessed as an effective therapeutic approach to relieve pain in patients with IBD.

## Methods

### Mice

C57BL/6, B6.Rosa26-stop(flox)-hM4Di [strain: B6N.129-*Gt(ROSA)26Sor*^*tm1(CAG-CHRM4*^*∗*^*,-mCitrine)Ute*^/J] and B6.Rosa26-GFP(flex)-hM3Di mice [strain: B6.Cg-*Gt(ROSA)26Sor*^*tm3.3(CAG-EGFP,-CHRM3*^*∗*^*/mCherry/Htr2a)Pjen*^/J] (Cat. No. 026219 and 026943 respectively) were purchased from Jackson Laboratory. The TRPV1-cre mice were provided by Dr G. Zamboni (University of Calgary) and bred at the University of Calgary Animal Resource Center. All mice were genotyped with the primers reported in Table 3. To study the inhibition of TRPV1 neurons, TRPV1-Cre+/− mice were bred with B6.Rosa26-stop(flox)-hM4Di+/+ mice to generate TRPV1 neurons expressing hM4Di (TRPV1-Cre+;hM4Di+ or TRPV1-hM4Di) mice and control (TRPV1-Cre-;hM4Di+ or hM4Di) littermates. To study the activation of TRPV1 neurons, TRPV1-Cre+/− mice were bred with B6.Rosa26-GFP(flex)-hM3Di+/+ mice to generate TRPV1 neurons expressing hM3Dq (TRPV1-Cre+;hM3Dq+ or TRPV1-hM3Dq) mice and control (TRPV1-Cre-;hM3Dq+ or hM3Dq) littermates. All mice were housed under standard conditions with drinking water and food available ad libitum. Age-matched 6- to 8-week-old littermate mice of both sexes were used in this study. All experiments were conducted according to the protocols approved by the University of Calgary Animal Care Committee and the guidelines of the Canadian Council on Animal Care.

**Table 3:** Primer Sequences

Genotype	Primer	Sequence 5'--> 3'	Primer type
hM4Di	22691	TCA TAG CGA TTG TGG GAT GA	Mutant reverse
	oIMR7882	CGA AGT TAT TAG GTC CCT CGA C	Mutant forward
	oIMR9020	AAG GGA GCT GCA GTG GAG TA	Wild type forward
	oIMR9021	CCG AAA ATC TGT GGG AAG TC	Wild type reverse
hM3Dq	28675	GGA GCA ACA TAG TTA AGA ATA CCA G	Mutant reverse
	28674	TGT ATC CAG GAG GAG CTG ATG	Mutant forward
	Oimr9020	AAG GGA GCT GCA GTG GAG TA	Wild type forward
	24500	CAG GAC AAC GCC CAC ACA	Wild type reverse
TRPV1-cre	oIMR1084	GCG GTC TGG CAG TAA AAA CTA TC	Mutant forward
	oIMR1085	GTG AAA CAG CAT TGC TGT CAC TT	Mutant reverse
	13922	TTC AGG GAG AAA CTG GAA GAA	Wild type forward
	13923	TAG TCC CAG CCA TCC AAA AG	Wild type reverse

TRPV1, Transient receptor potential vanilloid member 1.

### Induction of Colitis and Inflammation Assessment

Acute colonic inflammation was induced by administration of 2.5% (wt/vol) DSS (Alfa Aesar, Cat. No. J63606) in drinking water for 7 days. Visceral hypersensitivity post-colitis was induced as previously described.^15^ Briefly, colitis was induced by administration of 2.5% DSS (wt/vol) in drinking water for 5 days. On day 5, DSS was removed and replaced by water to allow mice to recover for 5 weeks. Body weight was monitored daily during DSS exposure and weekly during the recovery period. Macroscopic damage of the colon was assessed and scored based on the following parameters: adhesions (0, absent; 1, moderate; 2, severe); edema (0, absent; 1, moderate; 2, severe); strictures (0, absent; 1, 1; 2, 2; 3, >2); blood (0, absent; 1, present); ulcer (0, absent; 1, present); and mucus (0, absent; 1, present).

### Chemical Inhibition of TRPV1^+^ Nociceptors During Colitis

Six- to eight-week-old TRPV1-hM4Di and hM4Di littermate mice were injected intraperitoneally (i.p.) with 1 mg.kg of CNO (Tocris Bioscience, Cat. No. 4936) dissolved in saline twice daily for 7 days of DSS treatment. Vehicle-treated mice were injected i.p. with saline.

### Chemical Activation of TRPV1^+^ Nociceptors in Naive Condition

Six- to eight-week old TRPV1-hM3Dq and hM3Dq littermate mice were orally administrated by gavage with 0.1 mg.kg of CNO (Tocris Bioscience, Cat. No. 4936) dissolved in saline daily for 7 days. Vehicle-treated mice received by saline gavage.

### P2RY12 Antagonist Injection During Colitis

To assess the precise role of microglial P2RY12 in the spinal cord, DSS-treated mice were injected intrathecally with saline or dimethyl sulfoxide (Sigma-Aldrich) as control and the selective P2RY12 antagonist MRS2395 (Sigma-Aldrich, Cat. No. 491611-55-3) at day 2 and day 5 (25 μg in 10 μl in dimethyl sulfoxide) and once a week during the recovery period. Briefly, mice were anesthetized by isoflurane, the dorsal fur of each mouse was shaved, the spinal column was arched, and a 30-gauge needle attached in a PE20 polyethylene tube to a 25-μl Hamilton micro syringe was inserted into the subarachnoid space between the L4 and L5 vertebrae. Intrathecal injections of 10 μl were delivered over a period of 5 seconds.

### Visceromotor Response to Colorectal Distension

VMR to CRD was performed as previously described.^18^ Briefly, mice were anesthetized with xylazine/ketamine and implanted with 2 electrodes in the abdominal external oblique muscle. The mice were allowed to recover for 2 days before visceral sensitivity assessment. For recording, electrodes were connected to an electromyogram acquisition system via a Bio Amplifier (both from ADInstruments), and a 10.5-mm diameter balloon catheter (Edwards Life-Sciences, Cat. No. 12TLW404F) was inserted 5 mm proximal to the mouse rectum. Mice were subjected to four 10-second distensions (15, 30, 45, and 60 mmHg pressure) with 5-minute rest intervals. Electromyographic activity of the abdominal muscles was recorded, and VMR was calculated using LabChart 7 (ADInstruments).

### Immunohistochemistry of DRG Neurons

Animals were perfused with phosphate buffered saline (PBS) to wash out blood and then perfused with 4% paraformaldehyde (PFA) (Electron Microscopy Science, Cat. No. 15713). Thoracolumbar (T10–L1) and lumbosacral (L6–S1) DRGs were extracted and dehydrated overnight in 30% sucrose and embedded in optimal cutting temperature (OCT) solution (VWR International, Cat. No. 95057-838). Embedded tissues were sliced 10-μm thick in a serial fashion, and distributed over 4 Superfrost Plus slides (VWR International), ensuring that each individual slide contained sections that were at least 40 μm apart from each other and thus reduce the probability of double counting neurons.

To look at the proportion of hM4Di or hM3Dq receptor in peptidergic and non-peptidergic neurons, DRG sections were blocked for 1 hour in 5% bovine serum albumin (BSA) in 0.3% Triton X-100 at room temperature (RT). Subsequently, sections were incubated overnight at 4°C with primary antibody mix in 1% BSA in 0.02 % Triton X-100 (rabbit anti-TRPV1, 1:250, Alomone, Cat. No. ACC-030; rabbit anti-CGRP, 1:1000, Sigma-Aldrich, Cat. No.PC205L; goat anti-GFRα2, 1:500, R&D Systems, Cat. No. AF429; anti-IB4-coupled Alexa 594, 1:1000, Invitrogen, Cat. No. I21412; anti-HA, 1:500, Biolegend, Cat. No. 901502). Afterward, sections were washed in PBS and incubated with secondary antibody mix in 3% BSA in 0.02% Triton X-100 for 1 hour at RT (goat anti-rabbit Alexa 488, 1:2000, Invitrogen, Cat. No. A11008; chicken anti-rabbit Alexa 647, 1:2000, Invitrogen, Cat. No. A21443, chicken anti-goat Alexa 647, 1:2000, Invitrogen, Cat. No. A21469; goat anti-mouse Alexa 555, 1:3000, Invitrogen, Cat. No. A21422; goat anti-mouse Alexa 488, 1:2000, Invitrogen, Cat No. A11001).

To characterize the DREADD receptor in non-neuronal cells, after blocking in 5% BSA in 0.3 % Triton X-100, DRG sections were stained with a rat anti-F4/80 (1:500, Biolegend, Cat. No. 123102) or a rabbit anti-Connexin 43 (Cx43, 1:1000, Sigma-Aldrich, Cat. No. C6219) overnight at 4°C in 1% BSA in 0.02 % Triton X-100. Then, sections were incubated with secondary antibodies mix 3% BSA in 0.02% Triton X-100 (Donkey anti-rat Alexa 647, 1:2000, Cederlane, Cat. No. 712605153, chicken anti-rabbit Alexa 647, 1:2000, Invitrogen, Cat. No. A21443). All sections were mounted using Aqua PolyMount (Polysciences). Confocal images were acquired on a Zeiss LSM 510 Meta confocal microscope and AxioCam HRm camera and analyzed using a 20× objective. Sections were imaged and analyzed using ImageJ. A minimum of 3 mice per group were analyzed.

### Immunohistochemistry of Spinal Cord

Mice were perfused with PBS and then PFA 4%. Isolated thoracolumbar (T10–L1) and lumbosacral (L6–S1) spinal cord sections were dehydrated in 30% sucrose overnight and embedded in OCT solution (Thermo-Fisher Scientific). Embedded tissues were sectioned (10 μm) with 40-μm space between each section.^47^ This ensured that each individual section contained different cells and thus avoided counting cells multiple times. At least 3 sections per mouse, both right and left dorsal horn, were averaged, and means between animal groups were compared using appropriate statistical tests (see statistical analyses). A minimum of 3 mice per group were analyzed through 3 separated experiments. To assess dorsal horn neuron activation, cryosections were blocked for 1 hour in 5% BSA in 0.3% Triton X-100 at RT. Subsequently, sections were incubated overnight at RT with primary antibody mix in blocking buffer (c-Fos, 1:1000, Abcam, Cat. No. ab190289). Afterward, sections were washed in Tris buffered saline-Tween 20 and incubated with secondary antibody mix in 3% BSA in 0.02% Triton X-100 for 2 hours at RT (goat anti-rabbit Alexa 488, 1:500, Invitrogen, Cat. No. A11008 or chicken anti-rabbit Alexa 647, 1:1000, Invitrogen, Cat. No. A21443).

To quantify microglial activation, cryosections were blocked for 2 hours in 5% BSA in 0.3% Triton X-100 at RT. Subsequently, sections were incubated overnight at 4°C with primary antibody mix in blocking buffer (Iba-1, 1:1000, Fujifilm, Cat. No. 019-19741). Afterward, sections were washed in PBS and incubated with secondary antibody mix in 3% BSA in 0.02% Triton X-100 for 1 hour at RT (goat anti-rabbit Alexa 488, 1:500, Invitrogen Cat. No. A11008 or chicken anti-rabbit Alexa 647, 1:1000, Invitrogen, Cat. No. A21443). All sections were mounted using Aqua PolyMount (Polysciences). Confocal images were acquired on a Zeiss LSM 510 Meta confocal microscope and AxioCam HRm camera and analyzed using a 20× objective. Sections were imaged and analyzed using ImageJ. A minimum of 3 mice per group were analyzed through 3 separated experiments.

### Immunohistochemistry of Brain

After perfusion, isolated brain was dehydrated overnight in 30% sucrose and embedded in OCT solution (Thermo-Fisher Scientific). To characterize the DREADD receptor expression in the brain and assess if it overlaps with non-neuronal cells, 30-μm sections were blocked for 2 hours in 5% BSA in 0.3% Triton X-100 at RT. Subsequently, sections were incubated overnight at 4°C with primary antibody mix in blocking buffer (Iba-1, 1:1000, Fujifilm, Cat. No. 019-19741). Afterward, cryosections were washed in PBS, incubated with secondary antibody mix in 3% BSA in 0.02% Triton X-100 for 1 hour at RT (chicken anti-rabbit Alexa 647, 1:1000, Invitrogen, Cat. No. A21443) and mounted using Aqua PolyMount (Polysciences). Confocal images were acquired on a Zeiss LSM 510 Meta confocal microscope and AxioCam HRm camera and analyzed using a 10× objective. Sections were imaged and analyzed using ImageJ.

### Immunohistochemistry of Colon

Isolated colon was post-fixed in PFA 4% overnight, then incubated in 30% sucrose overnight and embedded in OCT solution (Thermo-Fisher Scientific). To determine the DREADD receptor in non-neuronal cells and in the ENS, 10-μm sections were blocked in 5% BSA in 1 % Triton X-100, and stained with a rabbit anti CGRP (1:1000, Sigma-Aldrich, Cat. No. PC205L) overnight at 4°C in 1% BSA in 0.3 % Triton X-100. Then, sections were incubated with secondary antibodies mix 1% BSA in 0.3 % Triton X-100 (chicken anti-rabbit Alexa 647, 1:2000, Invitrogen, Cat. No. A21443). All sections were mounted using Aqua PolyMount (Polysciences). Confocal images were acquired on a Zeiss LSM 510 Meta confocal microscope and AxioCam HRm camera and analyzed using a 40× objective. Sections were imaged and analyzed using ImageJ.

### Colonic Cytokine Profiling

Colonic samples were homogenized in RIPA buffer (1× PBS, 1% Igepal CA-630, 0.5% sodium deoxycholate, and 0.1% sodium dodecyl sulphate [all from Sigma-Aldrich]) containing Complete-Mini protease inhibitor (Thermo Fisher Scientific, Cat. No. 78430) and phosphatase inhibitor (Thermo Fisher Scientific, Cat. No. 78428). Lysates were centrifuged at 13,000 × g for 10 minutes at 4°C, and supernatants were collected. Samples were processed using a MILLIPLEX Mouse Cytokine Array Proinflammatory Focused 10-plex (Eve Technologies). Protein concentration was quantified using a Bradford assay (Bio-Rad Laboratories) for normalization.

### P2RY12 Expression

Spinal cords were homogenized using a bullet blender (Next Advance) with SSB02 beads (Next Advance) and lysed in RIPA buffer with protease and phosphatase inhibitors (Thermo Scientific) for 45 minutes. Lysates were centrifuged at 10,000g for 10 minutes at 4°C, supernatants were collected, and protein concentration was quantified and normalized using a Bradford assay (Bio-Rad laboratories). Total lysates were separated by sodium dodecyl sulphate–polyacrylamide gel electrophoresis (7.5%) and transferred onto nitrocellulose membranes (Sigma-Aldrich). Membranes were blocked in 5% nonfat dry milk for 1 hour at RT, and then probed with anti-P2RY12 antibody (1:200 dilution in 1% milk, Alomone, Cat. No. APR-012) at 4°C overnight. Membranes were then washed 3 times with Tris buffered saline-Tween 20 and incubated with horseradish peroxidase-conjugated anti-rabbit antibodies (1:1000; Cederlane, Cat. No. NA934) for 1 hour at RT. Bands were visualized using the Immobilon Western chemiluminescent HRP Substrate (Bio-Rad Laboratories), and band density was calculated using Image J. Intensity of Rabbit anti-Beta-tubulin III antibody (1:1000 dilution in 1% milk; Sigma-Aldrich, Cat. No. T2200) band was used for normalization among samples.

### DRG Neuron Isolation

DRG neurons were collected from 6- to 8-week old mice and enzymatically dissociated in Hank’s balanced salt solution containing 2 mg/mL collagenase type I and 4 mg/mL dispase (both from Invitrogen) for 45 minutes at 37 °C. DRGs were rinsed twice in Hank’s balanced salt solution and once in neurobasal A culture medium (Thermo Fisher Scientific) supplemented with 2% B-27, 10% heat-inactivated fetal bovine serum, 100 μg/mL streptomycin, 100 U/mL penicillin, 100 ng/mL of nerve growth factor, and 100 ng/mL glial cell-derived neurotrophic factor (all from Invitrogen). Individual neurons were dispersed by trituration through a fire-polished glass Pasteur pipette in 4 mL media. The neurons collected from the mice were cultured overnight at 37°C with 5% CO2 in 96% humidity on glass coverslips previously treated with 25% poly-ornithine and laminin (Sigma-Aldrich, Cat. No. P4957 and L2020, respectively) (for electrophysiological measurements and ATP release) and used the next morning.

### CNO-induced ATP Release

ATP levels were detected using bioluminescence by combining samples with recombinant firefly luciferase and its substrate d-luciferin (ATP Determination Kit, Life Technologies, Cat. No. A22066). The ATPase inhibitor (ARL67156, 30 μM, Sigma-Aldrich) was added to the medium to minimize breakdown of ATP. Samples were exposed to CNO (10 μM) for 5 minutes, then the supernatant was collected, and samples read immediately using a FilterMax F5 plate reader at 28°C. ATP measurements were expressed relatively to control samples.

### Electrophysiological Measurements

Current clamp experiments were performed using small diameter mouse DRG neurons at RT in a 2-mL bath containing (in mM): 140 NaCl, 5 KCl, 1.5 CaCl2, 2 MgCl2, 10 HEPES, and 10 D-glucose (pH 7.4 adjusted with NaOH, and osmolarity 315 mOsm). The small diameter neurons were identified via either mCitrine or mCherry fluorescence using an inverted epi-fluorescence microscope (Olympus IX51, Olympus America Inc). Borosilicate glass (Harvard Apparatus Ltd) pipettes were pulled and polished to 3- to 4-MΩ resistance with a DMZ-Universal Puller (Zeitz-Instruments GmbH). Pipettes were filled with an internal solution containing (in mM): 140.0 KCl, 5.00 NaCl, 1 CaCl2, 1.0 EGTA, 10.0 HEPES, 1.0 MgCl2, 3.0 MgATP, and 0.5 GTP (pH 7.3 adjusted with KOH, 315 mOsm). Recordings were performed using an Axopatch 200B amplifier (Axon Instruments). Current clamp protocols were applied using pClamp 10.5 software (Axon Instruments). Data were filtered at 5 kHz and digitized at 10 kHz with a Digidata 1550 A converter (Axon Instruments). Average cell capacitance for the DRG neurons was of 14.16 ± 0.85 pF for the wild type neurons and of 13.79 ± 0.87 pF for the neurons from transgenic animals. Only those cells that exhibited a stable voltage control throughout the recording and responded to capsaicin (100 nM for 10 seconds) challenge were used for analysis. The spontaneous activity of the DRG neurons was recorded at room temperature (∼ 22°C) for 3 minutes before application of the either capsaicin (100 nM for 10 seconds) or CNO (10 μM) (applied to the bath at ∼ 1000 μm from the cell at a rate of 500 μl/min). Only the neurons in which the resting membrane potential was more negative than -40 mV were used. All the drugs used (CNO 10 μm and capsaicin 100 nM [Sigma-Aldrich]) were diluted in the extracellular buffer, from a stock solution, to achieve final concentrations. All solutions were prepared and used at RT (22 ± 2ºC). The number of spontaneous APs was calculated as the number of APs per second during extracellular buffer application. The number of evoked APs was calculated as APs per second evoked by increasing injected currents. Data analysis and offline leak subtraction were completed in Clampfit 10.4 (Axon Instruments).

### Statistical Analyses

Statistical analyses were performed with GraphPad Prism 7 software. Normal distribution was verified using the D’Agostino-Pearson normality test. For Gaussian data, the Student *t* test was used to assess statistical significance when comparing 2 means, 1-way analysis of variance followed by the Tukey post hoc test was used to compare more than 2 groups and 2-way analysis of variance followed by Bonferroni (2 groups) and Tukey post hoc test (for more than 2 groups) for multiple comparisons. For non-Gaussian data, the non-parametric Mann-Whitney *U* test was used to assess statistical significance when comparing 2 means; the Kruskal-Wallis followed by the Dunn post hoc test was used to compare more than 2 groups. Statistical significance was established at *P* ≤ .05. Values were expressed as means ± standard error of the mean.
